# Supplementary material for: ‘Good health means being mentally, socially, emotionally and physically fit’: women’s understanding of health and ill health during and after pregnancy in India and Pakistan: a qualitative study
Source: BMJ Open. 2020 Jan 21;10(1):e028760. doi: 10.1136/bmjopen-2018-028760 (PMC7045203; doi:10.1136/bmjopen-2018-028760)
Supplement: Supplementary data [file bmjopen-2018-028760supp001.pdf]

LSTM
CENTRE FOR MATERNAL AND NEWBORN HEALTH

# Topic guides for women during and after pregnancy for their views and understanding of maternal health and ill-health

## 1. TOPIC GUIDE FOR PREGNANT AND POSTNATAL WOMEN

### PROFILE OF WOMEN

Once participants have signed the consent form, ask them to answer these following questions. It will help analyse the qualitative data.

Study setting: ____________________________________________

Age: ____________________________________________

Parity: ____________________________________________

Pregnant status / antenatal or postnatal and number of weeks: ______________________

### FORMAT

- Introduce yourself and welcome the group.
- Explain that we are here to learn more about the groups' views on their general views regarding what they understand all types of ill-health to be during and after pregnancy.
- In addition, explain that we are keen to understand what the group in general think regarding questions concerning other types of ill-health that may be a risk factor to pregnancy and how best to approach and ask these questions routinely in their setting.
- Ensure everyone has provided information for the registration log sheet and has a participant information sheet.
- Obtain informed consent for each participant.
- Provide a brief introduction to the relevant ground rules for the key informant interview or focus group discussion:
  (1) The discussion will last a maximum of 1 hour
  (2) The content of the discussion will remain confidential and will not be used outside of the group except for the purposes of the research
  (3) Participants are also requested to keep the discussion confidential after the session
  (4) Participants' names will not be used when reporting the findings
  (5) Participants are encouraged to speak their mind and there are no right or wrong answers
  (6) Participants should not hesitate to disagree with someone else
  (7) But should not talk at once
  (8) The interview will be recorded using a small hand held voice recorder device.
- A voice recorder will be used only to ensure that none of the information from the FGD is lost, and to facilitate analysis of the data from the interviews.

*Qualitative research: What is ill-health during and after pregnancy?*

## MATERIALS

Topic guide, log, notepad, pens, voice recorders, batteries, information sheet, consent form, transport reimbursement and reimbursement form, refreshments, voice recorders.

## FGD QUESTION GUIDE

Reminder to moderator: listen actively and always use probing questions to follow up and obtain further detail from the group (e.g. can you tell me more about that, can you give an example, why is that).

**Introduction**

We are very interested to hear about your general views regarding what you understand health and ill-health to be for women that you care for during and after pregnancy.

In addition, we are keen to understand how best to ask general questions concerning other types of ill-health that may be a risk factor to the pregnancy. These general questions will include how best to screen for psychological ill-health (depression) or social ill-health (domestic violence or substance misuse) during and after pregnancy.

We would like to understand your perceptions regarding the possible barriers or enabling factors to routinely screen women during and after pregnancy for all types of ill-health (physical, psychological and social) and whether you think women's general health needs are current being met in routine antenatal and postnatal care in your setting.

**General questions to ask**

1. What is health and what is ill-health during and after pregnancy?

We are interested to know about your general views of what health is and what ill-health in general is for women during and after their pregnancy.

- What do women in your setting consider to be 'good health'?
- What do women in your setting you consider to be 'poor health'?
- In relation to pregnancy: what would good health be and what would poor health be?
- If we consider poor health in pregnancy, what does this mean?
- What kinds of conditions or complaints do pregnant women tend to have?
- Is poor health different in pregnancy and after pregnancy? How?
- Probe: is health only related to physical health?

- Probe: does ill-health during and after pregnancy also include psychological, and social factors?
- Probe: In your opinion, do women have ill-health during and after their pregnancy? and if so what how does this bother them?

2. How best can a healthcare provider ask questions routinely regarding other forms of ill-health women experience during and after pregnancy?

We are keen to understand how best you think a healthcare provider can ask routinely regarding other forms of ill-health women experience during and after pregnancy, that may be a risk factor to the pregnancy, for example psychological ill-health (depression) or social ill-health (domestic violence or substance misuse)?

Can you tell us your general view and opinion on whether it is acceptable for healthcare providers to make enquiries regarding not just physical but also psychological and social ill-health during routine antenatal and postnatal care?

- Probe: What do you think about women being asked about their perception of their health in pregnancy?
- What kinds of questions do you think pregnant women should be asked to assess how they feel about their health?

- What do you think women would think regarding being asked about their mental well-being or social factors?
- Is it acceptable to be asked about this? Why? Why not?
- How do you think women should be asked these questions?

3. We would like to understand your general perceptions regarding the possible barriers or enabling factors to routinely screen women during and after pregnancy for all types of ill-health (physical, psychological and social).

    - In your community, how is pregnancy perceived?
    - Are pregnant women treated any differently? In what way?
    - When women are pregnant do they expect to feel ill?
    - What kinds of conditions or complaints are perceived as ‘routine’ during pregnancy?
    - What conditions or complaints are not considered a ‘routine’ part of pregnancy?
    - How do women deal with minor common discomforts s during pregnancy?

- How do women deal with those that are not considered a common part of pregnancy?

4. Do you think women's general health needs are current being met in routine antenatal and postnatal care in your setting?

We would like to understand more about whether the existing health care system in your general opinion, meets the health needs of women during and after pregnancy in your community?

Probe: To what extent do you think the health facility meets womens' needs during and after pregnancy? In your opinion does the health system in general provide good quality to women during and after pregnancy?

Probe: What do you think should be done to improve provision of services for women during and after pregnancy?

**In summary**:
**Ask if participants would like to add further comments.**

**Bring the meeting to a close by summarizing the main points.**

**Do not forget to say thank you to the participants for their time and active participation.**



McCauley M, *et al. BMJ Open* 2020; 10:e028760. doi: 10.1136/bmjopen-2018-028760
