# Supplementary material for: ‘Good health means being mentally, socially, emotionally and physically fit’: women’s understanding of health and ill health during and after pregnancy in India and Pakistan: a qualitative study
Source: BMJ Open. 2020 Jan 21;10(1):e028760. doi: 10.1136/bmjopen-2018-028760 (PMC7045203; doi:10.1136/bmjopen-2018-028760)
Supplement: Author's manuscript [file bmjopen-2018-028760.draft_revisions.pdf]

BMJ Open is committed to open peer review. As part of this commitment we make the peer review history of every article we publish publicly available.

When an article is published we post the peer reviewers' comments and the authors' responses online. We also post the versions of the paper that were used during peer review. These are the versions that the peer review comments apply to.

The versions of the paper that follow are the versions that were submitted during the peer review process. They are not the versions of record or the final published versions. They should not be cited or distributed as the published version of this manuscript.

BMJ Open is an open access journal and the full, final, typeset and author-corrected version of record of the manuscript is available on our site with no access controls, subscription charges or pay-per-view fees (http://bmjopen.bmj.com).

If you have any questions on BMJ Open's open peer review process please email info.bmjopen@bmj.com

BMJ Open

"Good health means being mentally, socially, emotionally and physically fit": Women's understanding of health and ill-health during and after pregnancy

Journal:	*BMJ Open*
Manuscript ID	bmjopen-2018-028760
Article Type:	Research
Date Submitted by the Author:	04-Jan-2019

Complete List of Authors:	McCauley, Mary; Liverpool School of Tropical Medicine, Centre for Maternal and Newborn Health<br>Avais, Ayesha ; Child Advocacy International Pakistan<br>Argawal, Ritu; Vardhman Mahavir Medical College and Safdarjung Hospital<br>Saleem, Shumaila; Child Advocacy International Pakistan<br>Zafar, S; Fazaia Medical College<br>van den Broek, Nynke; Liverpool School of Tropical Medicine, Centre for Maternal and Newborn Health

Keywords:	Maternal medicine < OBSTETRICS, Antenatal care, Postnatal care, Maternal morbidity, Understanding of health and ill-health

SCHOLARONE™
Manuscripts

"Good health means being mentally, socially, emotionally and physically fit": Women's understanding of health and ill-health during and after pregnancy

Authors:

MCCAULEY, Mary[1]*

AVAIS, Ayesha Rasheeda[2]

AGRAWAL, Ritu [3]

SALEEM, Shumalia[2]

ZAFAR, Shamsa[4]

VAN DEN BROEK, Nynke[1]

[1]Centre for Maternal and Newborn Health, Liverpool School of Tropical Medicine, United Kingdom

[2]Child Advocacy International Pakistan, Islamabad, Pakistan

[3]Safdarjung Hospital, New Delhi, India

[4]Fazaia-Medical College, Air University, Islamabad, Pakistan

*Corresponding author: mary.mccauley@lstmed.ac.uk

Word count: 4708

Ethical Approval: The Liverpool School of Tropical Medicine, Liverpool, United Kingdom, Safdarjung Hospital, New Delhi and the National Bioethics Committee, Islamabad, Pakistan all granted ethical approval.

Disclosure of Funding Received: This study was funded by a Research Development Fund from the Liverpool School of Tropical Medicine, Liverpool, United Kingdom.

Précis (Max 25 words)

Women in India and Pakistan recognise that ill-health is not just related to physical disease. Care packages need to be modified to address psycho-social maternal morbidity.

Short Title:

Women's views on health and ill-health during and after pregnancy in India and Pakistan.

Keywords:

Health, ill-health, antenatal care, postnatal care, pregnancy, maternal morbidity.

For peer review only - http://bmjopen.bmj.com/site/about/guidelines.xhtml

Article summary

Strengths and limitations of this study

• Maternal ill-health is an international public health concern. Many women experience ill-health during and after pregnancy, but currently comprehensive and systematic assessment of all components of health (physical, psychological and social) is not an established part of routine maternity care in many low- and middle-income countries.

• To the best of our knowledge, this is the first study from two LMIC settings to obtain women's own opinions on what they might consider health and ill-health to be in general and during and after pregnancy.

• A range of women from different stages of pregnancy were interviewed resulting in a wide spectrum of responses.

• This study population included women attending for routine antenatal or postnatal care in public secondary level healthcare facilities in urban settings in India and Pakistan and excludes other women who may have alternative perspectives or different insights.

• Our findings indicate that most women would welcome a psycho-social assessment during routine antenatal and/or postnatal care. Maternity care packages need to be modified to address all aspects of maternal ill-health, including psycho-social wellbeing.

For peer review only

Abstract

Objective

To explore what women, consider health and ill-health to be, in general, and during and after pregnancy. Women's views on how to approach and discuss all components of ill-health as part of routine screening during and after pregnancy, including psycho-social morbidity were also explored.

Setting

A public hospital in New Delhi, India and a public hospital in Islamabad, Pakistan.

Participants

130 women attending for routine antenatal or postnatal care at the study healthcare facilities.

Interventions

Data collection was conducted using focus group discussions and key informant interviews. Transcribed interviews coded by topic and grouped into categories. Thematic framework analysis was undertaken to identify emerging themes.

Results

Most women are aware that health is a multidimensional concept, not simply physical symptoms or identified medical disease. Concepts of "good health" included a nutritious diet; having an ideal weight; absence of disease; and a supportive family environment. Ill-health consisted of physical symptoms and disease; stress and tension; and domestic violence and alcohol use in the family. Women are aware that the health or ill-health of the mother and baby are interlinked. In addition to a full physical assessment, most women welcome psycho-social ill-health enquiries. Reported barriers to routine enquiry regarding psycho-social aspects of maternal health included women's perceptions that these issues are "personal"; that healthcare provider's do not have the time and/or cannot provide further care, even if psycho-social morbidity is disclosed.

For peer review only

Conclusions

Women have a good understanding of the comprehensive nature of health and ill-health during and after pregnancy. Women report that enquiry regarding psycho-social ill-health is not part of routine maternity care, but most welcome such an assessment.

Keywords

Health, ill-health, maternal health, women, antenatal care, postnatal care, qualitative research, perceptions.

Word count: 268

For peer review only

For peer review only - http://bmjopen.bmj.com/site/about/guidelines.xhtml

Introduction

All women have the right to the highest attainable standard of health and well-being including physical, psychological and social aspects [1]. The Sustainable Development Goals (SDG) envision that that every woman in every setting has an equal chance to survive and thrive [2]. SDG three is to ‘ensure healthy lives and promote well-being for all’ [2]. The Global Strategy strives for a world in which every mother can enjoy a wanted and healthy pregnancy and childbirth and that pregnancy is an opportunity for a woman to receive care to “thrive” [1]. Currently this is not the case in many low-and middle- income countries (LMIC). Although, the main priority is to ensure that no woman dies during or after pregnancy, the SDG also recognises the need to emphasis health and therefore, global maternal health targets and indicators have been expanded from a focus on maternal mortality, to emphasize the importance of well-being [2].

The World Health Organization (WHO) defines health as ‘a state of complete physical, mental and social well-being and not merely the absence of disease or infirmity’ [3]. It is imperative that healthcare providers are supported to provide good quality care for women and that this goes beyond simply the physical aspects of health and is inclusive of psycho-social well-being [4,5].

As part of the need to develop innovative strategies to achieve SDG 3, there has been a renewed focus on maternal ill-health or “maternal morbidity” in terms of definition, measurement, and interventions [4,6-8]. The burden of disease of the various aspects of health (physical, psychological and social) during and after pregnancy and their impact on women and/or pregnancy outcomes are currently not well documented in a comprehensive, standardised approach [4,6]. It is suggested that although maternal ill-health occurs across all countries, cultures, religions, socioeconomic status, and ages, the burden of maternal morbidity is expected to be higher in LMIC with an estimated three out of four women suffering at least one form of maternal morbidity during or after their pregnancy [4,6].

Maternal ill-health or morbidity is a complex and broad concept and can be understood and described in various ways depending on different perspectives. For example, women, men,

For peer review only - http://bmjopen.bmj.com/site/about/guidelines.xhtml

families, communities, healthcare providers and policy makers may have mixed views and perceptions on what maternal ill-health is and the effect or risk of maternal morbidity during and after pregnancy for the women and/or her baby. Maternal morbidity has been defined as "any health condition that is attributed to or aggravated by pregnancy and childbirth which has a negative impact on the woman's wellbeing" [9]. However, there is still a lack of understanding regarding what women themselves consider ill-health to be and their experiences (subjective measures) compared to clinical findings (objective measures) when assessed and defined as "morbidity" by a healthcare provider [4,6].

This study sought to explore what women attending for routine ANC and PNC at healthcare facilities in India and Pakistan consider health and ill-health to be in general, and, what they consider health and ill-health to be, during and after pregnancy. In addition, women's views on how best to approach psycho-social morbidity, were assessed.

Materials

Study design and setting

Experienced female researchers (AA, RA, SS) interviewed a total of 130 women. Data collection was conducted using focus group discussions (FGD) to interview 60 women attending for routine ANC or PNC in the outpatient clinics at a large public hospital in New Delhi, India. FDG and key informant interviews (KII) were used to interview 70 women attending for routine ANC or PNC in the outpatient clinics at a large public hospital in Islamabad, Pakistan. All interviews and discussions were held in quiet locations away from the clinical areas at the relevant hospitals.

Participants

All women attending for routine ANC or PNC in the study healthcare facilities were invited to participate. Similar proportions of women were included from four different stages of pregnancy to include the first and second half of pregnancy, early postnatal and late postnatal stages. Women were purposively selected and recruited sequentially until the number of anticipated FGD and KII was reached and saturation was achieved.

For peer review only - http://bmjopen.bmj.com/site/about/guidelines.xhtml

Topic guide

A topic guide was developed to guide the interviews and was piloted in the United Kingdom with clinical researchers from LMIC. The topic guide was then translated into the local study setting language, back-translation was conducted and the topic guide was piloted in each study setting. As a result, the topic guide was refined; for example, some wording was changed in the local language and the introduction was amended to ensure that the women were aware that we sought to assess their general views and not their own personal experiences (if any) of different types of ill-health. The topic guide served as a flexible tool to facilitate the facilitators in obtaining the women's opinions whilst ensuring that the interview remained on topic. The topic guide also acted as a cue to ask more probing questions to further understand women' beliefs and perceptions. The topic guide included three main subject areas:

1) perception and understanding of health and ill-health in general;

2) perception and understanding of health and ill-health during and after pregnancy;

3) views on enquiry regarding psycho-social morbidity during and after pregnancy; and suggestions on how best to approach all components of health.

Data Collection

Prior to interview, all eligible women were approached and given verbal and written information regarding the study including a brief overview of the research aims and interview questions. Appointments for FGD or KII were then scheduled for a later time convenient for the women. The FGD and KII were conducted in the local language by local researchers (India-Hindi and Pakistan-Urdu). The FGDs lasted 60-90 minutes, the KIIs lasted 30 minutes. All FGD and KII were conducted face-to-face, recorded on a digital recording device and transcribed in the local language upon completion by the local researchers (AA, RA, SS). All efforts were made to emphasise confidentiality for interviews to ensure women were confident in giving honest answers and member checking was used to assure correct interpretation. Interviewing women from different stages of pregnancy, different backgrounds, and different countries broadened the scope of the topic and enabled transferability to be achieved to an extent.

Analysis

For peer review only - http://bmjopen.bmj.com/site/about/guidelines.xhtml

In each study setting, transcribed interviews were translated into English and initially open coded following data familiarisation (reading and re-reading of the transcripts) by the local researchers (RA in India; AA and SS in Pakistan). The transcripts were then reviewed by a second researcher (MMc) for sense checking and to avoid bias. Using thematic analysis, codes were identified and grouped into categories independently by two researchers, enabling the first abstraction of data in each setting [10]. Thematic framework analysis of the categories was then undertaken. The separate results were then brought together and refined to agree on the key themes. This strengthened the results and helped to remove potential bias [10]. The reporting of the results adheres to the COREQ guidelines [11].

Results

A total of 130 women were interviewed (**Table 1**). Three women invited to participate declined due to time constraints. A total of 83 women were pregnant at the time of interview and 47 had recently given birth. Most women were either in their first pregnancy or had one previous pregnancy. A minority of women had more than two previous pregnancies. The age range was from 20-36 with a mean of 26 years old.

Table 1. Participants

**Stage of pregnancy**	**India**		**Pakistan**		**Total number of participants**
	**Method**	**Number of women**	**Method**	**Number of women**	
First half of pregnancy (≤20 weeks)	2 FGD	16	2 FGD	26	42
Second half of pregnancy (>20 weeks)	3 FGD	17	2 FGD	24	41
Early postnatal stage (day 1-7)	3 FGD	22	10 KII	10	32
Late postnatal stage (week 2-6)	1 FGD	5	10 KII	10	15
**TOTAL**		**60**		**70**	**130**

For peer review only - http://bmjopen.bmj.com/site/about/guidelines.xhtml

What is health?

Women perceived "good health" in general as having a good diet; having an ideal weight; absence of physical disease; absence of stress and tension; and a supportive family environment**.**

Most women expressed that the ability to have a good diet would result in good health.

> "Good health is from a good diet." (FGD 1, ANC, Pakistan)
>
> "If a person is happy but not eating well, how can she be healthy? Diet is so important." (FGD 1, ANC, Pakistan)
>
> "Good health is good food." (KII 6, PNC, Pakistan)
>
> "Health means to have balanced diet." (FGD 5, ANC, India)

The matter of "ideal weight" was mentioned in Pakistan. However, women's perception of this differed.

> "One should be strong and a bit bulky." (FGD 1, ANC, India)
>
> "Healthy women are those who are slim and have strength." (KII 3, PNC, Pakistan)

In both settings, women reported that health meant lack of disease and good energy levels.

> "Good health is when there is no disease, no deficiency." (FGD 3, ANC, Pakistan)
>
> "We should have the energy to do the chores, that means we are healthy." (FGD 4, ANC, Pakistan)

However, most women reported that health was not just physical health but includes mental health. Many women recognised the interaction between physical and mental health and reported that relieving stress and tension were important for overall health.

> "Being healthy means having no stress or worries and having a tension-free life." (FGD 5, ANC, India)
>
> "There is health of the mind and other body parts. If one body part is missing or is not healthy, one cannot be considered healthy. Similarly, if one is not mentally healthy, one is not able to do anything." (FGD 1, ANC, Pakistan)
>
> "Good health means being mentally, socially, emotionally and physically fit." (FGD 2, ANC, India)

For peer review only - http://bmjopen.bmj.com/site/about/guidelines.xhtml

Most women highlighted that a supportive environment with family were important for good health.

> "The surrounding environment and family atmosphere directly affects health." (FGD 4, PNC, India)

> "If there is peace in the home and in the mind, then it is good health." (KII 3, PNC, Pakistan)

> "If there are fights in the home, this effects a woman's health negatively." (KII 8, PNC, Pakistan)

What is health during and after pregnancy?

During and after pregnancy a nutritious diet; rest; extra self-care; and a peaceful home and family environment were mentioned as specifically important. Most women reported that diet is even more important when a woman is pregnant as it is important to provide enough nutrients for both the mother and baby.

> "In pregnancy, dietary care is more important to remain healthy, as there is a baby as well." (FGD 4, ANC, India)

> "One needs to be extra careful and eat more in pregnancy because it is the matter of two lives now." (KII 2, PNC, Pakistan)

A minority of women felt that health in pregnancy was the same as health in general.

> "It (health in pregnancy) is same as in routine; like if a person is healthy, fit and has no fear or issue, and has no disease, [then] health is good." (KII 4, PNC, Pakistan)

Many women mentioned that pregnant women needed extra care including looking after themselves more than before and needed more care from the family and healthcare providers.

> "Good health in pregnancy is when you take your medicine regularly and completely." (KII 4, PNC, Pakistan)

> "You have to take really good care of yourself in pregnancy. If you become weak, the complications increase and the baby will also be weak." (KII 4, PNC, Pakistan)

For peer review only - http://bmjopen.bmj.com/site/about/guidelines.xhtml

> "Pregnant woman must take a nap for one and a half hour to two hours during daytime and try to take more rest at night." (FGD 2, PNC, India)

> "Women should not have any tension during pregnancy." (FDG 3, ANC, India)

Many women understood that their health (or ill-health) was linked to that of their baby.

> "The most important thing is this that baby is healthy and to have the proper medical care so that both baby and mother stay healthy." (KII 7, PNC, Pakistan)

> "If family members are quarrelsome and abusive, it affects both the mother and baby's health." (FGD 2, ANC, India)

What is ill-health?

The emerging themes related to perceived ill-health included; physical symptoms and disease; tension; and an abusive and stressful home environment.

Many women expressed that ill-health was related to physical symptoms such as pain, flu, fever, sore throat, weakness, body ache, headache, and swelling. Ill-health was also described in terms of disease, for example HIV/AIDS.

> "Those who are not healthy, their face shows it; they have yellowish complexion and have dark circles around the eyes." (FGD 1, ANC, Pakistan)

> "Ill-health means presence of disease." (FGD 4, ANC, India)

> "Ill-health is like flu, fever and sore throats." (KII 5, PNC, Pakistan)

> "Poor health means having pain, discomfort, fever and body ache." (KII 4, PNC, Pakistan)

> "Disease is for example HIV/AIDS, weak bones, weakness." (KII 5, PNC, Pakistan)

Mental ill-health was often considered a health concern and stress and tension was reported by many women as a component of or factor leading to mental ill-health.

> "One should be mentally healthy. Health is not only physical." (FGD 3, ANC, India)

> "Mental health affects physical health. If one is mentally disturbed, won't be able to do anything, even if you are physically fit, you will feel weak." (KII 9, PNC, Pakistan)

> "Ill-health is about tension; even if there is a very low level of stress or tension it does great damage to a person." (KII 2, PNC, Pakistan)

For peer review only - http://bmjopen.bmj.com/site/about/guidelines.xhtml

What is ill-health during and after pregnancy?

Perceived ill-health during and after pregnancy included the perception that pregnancy is a "disease", and that there was "no health in pregnancy", as all "the energy goes to the baby and the mother becomes weaker". Women also considered some physical symptoms and discomfort as "normal" during pregnancy. This included nausea and vomiting, back pain and difficulty sleeping.

> "There are normal issues… they are normal in pregnancy." (KII 7, PNC, Pakistan)

However, many women reported that ill-health during and after pregnancy was because of physical symptoms such as vomiting, high blood pressure, bleeding, swelling, abdominal pain. Many women were aware that these symptoms could be dangerous.

> "Ill-health is …. abdominal pain, leaking per vagina, vomiting, loss of appetite and dizziness." (FGD 2, ANC, India)

> "There are many symptoms that are dangerous, like if there is lack of fluids or baby around the baby is not growing as it should be, or if baby is suffering due to negligence." (KII 5, PNC, Pakistan)

> "Bleeding or swelling is dangerous." (FGD 3, ANC, Pakistan)

Stress and tension were considered important components of ill-health by most women with some discussion about examples and consequences during and after pregnancy.

> "Stress affects health in pregnancy, if there is any stress or tension one cannot eat properly. It also affects the baby's health and our own health too." (KII 4, PNC, Pakistan)

An abusive family environment including domestic and alcohol abuse from the husband and/or family members were highlighted as examples of social ill-health in women during and after pregnancy.

> "If family members are quarrelsome and abusive, it affects both the mother and baby's health." (KII 7, PNC, Pakistan)

> "The husband comes home after drinking and beats the woman. They don't even see that she is pregnant and she can get hurt and anything can happen." (FDG 2, ANC, India)

> "If a husband doesn't even earn much and spends his entire income on drinking, he comes home late and beats his pregnant wife." (FDG 4, ANC, India)

For peer review only - http://bmjopen.bmj.com/site/about/guidelines.xhtml

There was some discussion regarding gender preference and it was suggested by a minority of women that the gender of a baby created stress and tension for women during and after pregnancy.

> "In some families, male gender preference is a major cause of stress and tension for pregnant women." (FDG 1, ANC, India)

> "This time also I have given birth to a girl child [and] because of this the family atmosphere is not good. My family members are abusing me and because of this I am going through a lot of stress." (FDG 5, PNC, India)

Finally, women were worried about the health of their unborn baby if their own health was no optimum or health needs were not met.

> "When I do not feel physically well in pregnancy, I feel stressed and scared that this might affect my baby, like if there is deficiency of folic acid, the baby will be crippled/disabled. This gives stress in pregnancy." (KII 2, PNC, Pakistan)

Should healthcare providers ask about psycho-social ill-health?

An emerging theme regarding what women consider health and ill-health to be, was the importance of psycho-social well-being. Women described psychological ill-health such as "depression", "weeping", stress", "tension", "grief", "loss of appetite" and "unhappiness". Women reported social concerns such as "fights in the home", "domestic violence", "poor family environment", "quarrels and verbal abuse", "family or husband alcohol misuse", and an "expectation of a male child". Women were therefore asked questions regarding how best to approach and address the concept of psycho-social ill-health for women during and after pregnancy. Many women reported that healthcare providers did not enquiry regarding their psycho-social well-being. Women gave suggestions of how healthcare providers could approach such issues; and discussed barriers and solutions regarding such questions.

Most women reported that healthcare providers ask pregnant women questions regarding physical ill-health only.

> "They do not ask about mental health or the family situation. They just ask about test reports, blood deficiency and so on" (FGD 4, ANC, Pakistan)

> "They ask in detail about me, about my diet and medicines, they take a complete history and ask about all my previous deliveries but they never ask about my family environment or mental health." (KII 10, PNC, Pakistan)

For peer review only - http://bmjopen.bmj.com/site/about/guidelines.xhtml

> "They usually don't ask much, and we also do not talk much; they check, give medicine, or sometimes ask, does your husband smoke and is there any disease in the family?" (KII 7, PNC, Pakistan)

Most women felt that there was a lack of enquiry from healthcare providers regarding psycho-social ill-health during and after pregnancy and only one woman mentioned that her healthcare provider had asked whether "there was any tension?".

Most women felt that healthcare providers could and should routinely ask women about their psycho-social health during routine antenatal and postnatal visits.

> "Yes, they should ask us." (FGD 4, ANC, India)

> "They should ask such questions, but they do not. They should ask about family conflicts and talk about the solutions." (KII 11, PNC, Pakistan).

Many women said that it would be beneficial to speak to their healthcare provider if they were feeling "stressed" and "under tension" as the healthcare provider might be able to provide support and advice.

> "If one has some grief, sharing with someone lessens the grief, though it may not solve the problem, but releases stress." (KII 6, PNC, Pakistan)

However, a few women explained these types of questions might be considered unacceptable, because of lack of education and understanding among some women.

> "Women who are uneducated or unaware might get offended by these questions, others will not mind." (FGD 2, ANC, Pakistan)

> "People who understand such issues will not react but others will." (FGD 3, ANC, India)

There was some concern that some women would prefer not to talk about their psycho-social ill-health as they would be afraid, ashamed or uncomfortable.

> "Some women will not talk about their problems as they are afraid. They think that their husband will know about it if they talk to doctor and will be angry with them." (FGD 4, ANC, Pakistan)

> "I will feel ashamed to talk about my condition… I can tolerate till it is really necessary." (KII 8, PNC, Pakistan)

> "Some women don't share. Some women keep everything inside. It also depends upon the woman's personality." (KII 11, PNC, Pakistan)

For peer review only - http://bmjopen.bmj.com/site/about/guidelines.xhtml

A small minority of women in Pakistan felt it was not the role of the healthcare provider to ask questions regarding psycho-social health, as this was their “personal life”.

> “Doctors should not ask these questions, no, they should not.” (KII 5, PNC, Pakistan)

> “No, doctor has no right to….no doctor has no right to ask such questions.” (KII 6, PNC, Pakistan)

Women discussed the repercussions from husbands and family if domestic violence during and after pregnancy would be disclosed by a woman to a healthcare provider. There were concerns that a woman would not be allowed to access care again. There was a lot of discussion about this with sometimes conflicting opinions; ranging from it not being right for a woman to disclose domestic violence, or psychological ill-health such as stress and tension; to a consideration that healthcare providers should ask and would provide help.

> “If you ask about the family situation, our community is such that women will be afraid as to why these questions are being asked and they have a fear that in-laws will find out” (FGD 2, ANC, Pakistan)

> “If the doctor asks in front of our family, that will help change bad behaviours in the family” (FGD 3, ANC, Pakistan)

A few women said that if they themselves were asked questions regarding depression or domestic violence, they would seek care from another healthcare provider to avoid these questions.

Many women recognised that there were existing barriers for healthcare providers which made it difficult for them to ask women questions regarding psycho-social ill-health during and after pregnancy. Such barriers included the perceived heavy workload of healthcare providers and lack of time to explore psycho-social concerns.

> “A doctor has many patients and if she goes in this much detail with just one patient what about the other patients? Obviously, they have so much burden they don’t have time for these details!” (FGD 4, ANC, Pakistan)

Other women explained that “they can ask, but what can they do?” and that “nothing can be done” to help. Some women did offer suggestions of how healthcare providers could best ask questions regarding psycho-social ill-health during and after pregnancy. A more empathic approach to women were suggested including politeness and friendliness were suggested. Confidentiality was highlighted as important by many women.

For peer review only - http://bmjopen.bmj.com/site/about/guidelines.xhtml

"Though these are personal matters, but still they can ask such [social health] questions in private but just to understand patient's condition better like why her BP is high and is she going through any problem?" (FGD 5, ANC, Pakistan)

Some women suggested that healthcare providers need not go into such detailed questions but keep any questions simple.

"They should use questions in a way that are focused and clear." (FGD 3, ANC, Pakistan)

"Do you have any tension? How is your home environment? How is your husband and your family-in-laws behaviour with you?" (KII 10, PNC, Pakistan)

"You could ask, how does your husband treat you?" (FGD 4, ANC, India)

"How are you? How is everyone else in the family? Do you live in a joint family system? So, in this way, a woman can start talking about her pain and grief." (FGD 3, ANC, Pakistan)

Discussion

Statement of principal findings

Most women who took part in this study had clear opinions and had a good understanding that health includes more than physical components of health and that the health of the mother was linked to that of the baby. Good diet, healthy weight, rest and a supportive family living environment contributed to good health; whereas physical disease symptoms, complications of pregnancy, as well as stress, tension and an abusive family environment contributed to ill-health. Women were aware of physical common discomforts and of more severe complications and dangers signs, as ill-health related to pregnancy. Women considered psycho-social ill-health as important but reported that healthcare providers do not routinely ask women how they are feeling or ask after their psycho-social health. However, most women would be happy if healthcare providers did so. A minority of women considered psycho-social concerns as "personal", and there may be barriers to asking such questions.

Strengths of the study

To the best of our knowledge, this is the first study from a LMIC setting to obtain women's own opinions on what they might consider health and ill-health to be in general and during and after pregnancy. A range of women from different stages of pregnancy were interviewed

For peer review only - http://bmjopen.bmj.com/site/about/guidelines.xhtml

resulting in a wide spectrum of responses. Most women approached welcomed the discussion surrounding health, and ill-health during and after pregnancy, and were keen to contribute to solutions in their settings regarding healthcare providers roles in enquiry regarding other components of health, and not just physical health.

Limitations of the study

This study population included women attending for routine antenatal or postnatal care in public secondary level healthcare facilities in urban settings and excludes other women who may have alternative perspectives or different insights. There is a need to assess the views of women in other LMIC and including community and rural settings, as these women may have different perceptions and understandings.

How does this study relate to other literature?

There is lack of literature regarding the understanding of what women consider health and ill-health to be during and after pregnancy in India and Pakistan. There is also debate that the current definition of health [3] is out-dated and needs to be re-formulated to consider health in a context of functionality, capacity, adaptability and the ability to perform activities of daily living despite having a disease or disability [12]. There have been various proposals to develop the definition of health using the concept of “health, as the ability to adapt and to self-manage” but with a continued emphasis on the importance of the three domains of health: physical, psychological, and social [12].There have been calls for research into the understanding of health and ill-health during and after pregnancy as perceived by women themselves and to use this information to develop interventions and deliver maternity care in a way that meets the health needs of women in a comprehensive and holistic manner [4, 6].

Aspects of physical ill-health in women during and after pregnancy in LMIC settings are well documented and there are many recommendations and interventions developed to diagnose and manage these conditions; for example, anaemia, haemorrhage, hypertensive disorders of pregnancy, infection, and complications of obstructed labour [13-17]. With regards to psycho-social ill-health in women during and after pregnancy in LMIC, there is data to

For peer review only - http://bmjopen.bmj.com/site/about/guidelines.xhtml

demonstrate that depression and domestic violence represent a large burden of ill-health and have a negative impact on a woman's health and well-being [18-21].

However, to date, clinical practice in low resource settings rarely takes psycho-social ill-health into account when assessing a woman's health needs during and after pregnancy. This may be due to a shortage of human resources but also because of healthcare providers' perceptions that these issues are not "ill-health" per se and because of perceived cultural sensitivities related to psycho-social issues [4,5,23]. Although psycho-social ill-health is clearly a real matter of concern for women, this study was not designed to explore in further detail, as at the time we were not sure that women considered these components as "ill-health". Further research is needed to explain in more depth what these problems are and who and how they can be addressed.

It is well recognised that maternal physical and psycho-social ill-health is linked to newborn babies' health and women in our study demonstrated an understanding that all aspects of ill-health are associated with poor fetal and newborn health outcomes [24,25,26]. The detrimental impact of psycho-social morbidity on the overall health and well-being of mothers (and their babies) during and after pregnancy has resulted in public health policy in high income countries such as the United Kingdom (UK), where enquiry regarding of psycho-social ill-health including depression and domestic violence, is routinely conducted during and after pregnancy [27]. The WHO have produced clinical and policy guidelines on how to comprehensively care for women during and after pregnancy including identification, safety assessment and planning, communication and clinical skills, documentation and provision of referral pathways for psycho-social ill-health [28]. However, the feasibility of implementation and acceptability of this guidance in countries, such as India and Pakistan are currently uncertain. There is debate as to the cadre of healthcare providers most suitable to undertake routine enquiry regarding for and/or manage psycho-social ill-health in LMIC [5, 29]. In many high-income countries, specially trained midwives routinely assess, screen, support and provide further referral for women with psycho-social morbidity [27]. This study shows that most women are keen to be asked questions regarding their psycho-social well-being during and after pregnancy, as part of routine maternity care.

For peer review only - http://bmjopen.bmj.com/site/about/guidelines.xhtml

Currently, there is good coverage and uptake of antenatal care but content needs to be adapted to ensure good quality comprehensive care that is respectful, integrated and delivers physical, psycho-social care, with agreed country specific additional content. Although, psycho-social ill-health is clearly a real matter of concern for women, this study was not designed to explore this in further detail. Further research is needed to explain in more depth what these problems are, and who and how they can be addressed.

Conclusion

Women reported that health and ill-health is not just physical but also includes psychological and social components. Maternal ill-health is common among women attending for antenatal and postnatal care [4]. Women are increasingly accessing care during and after pregnancy and there is a window of opportunity to adapt and amend available care packages to include comprehensive enquiry regarding and, where needed, support for psycho-social ill-health [4, 11]. This study helps to give a better understanding of what women consider to be health and ill-health. This study demonstrates that most women are keen to be asked questions regarding their psycho-social well-being and provides simple suggestions that healthcare providers can use to improve the enquiry regarding psycho-social ill-health among women during and after pregnancy.

Abbreviations

HIC: High income country, **FGD**: Focus group discussion, **KII**: Key informant interviews, **LMIC**: Low and middle-income country, **LSTM**: Liverpool School of Tropical Medicine, **SDG**: Sustainable Development Goals, **UK**: United Kingdom, **WHO**: World Health Organization

Declarations

Ethics approval and consent to participate

The Liverpool School of Tropical Medicine (16-054), Liverpool, United Kingdom, Safdarjung Hospital (IEC/SJH/VMMC/Project/October-16/19/688), New Delhi, and the National Bioethics Committee (4-87/16/NBC-223), Islamabad, Pakistan, granted ethical approval. All women provided written, informed consent.

For peer review only - http://bmjopen.bmj.com/site/about/guidelines.xhtml

For peer review only

Consent for publication

Written informed consent was obtained from all women in the study, including audio-recording and the use of excerpts in publications and reports.

Availability of data and material

The datasets used and analysed during the current study are available from the corresponding author on reasonable request.

Competing interests

The authors declare that they have no competing interests.

Funding

Disclosure of funding received: This study was funded by the Liverpool School of Tropical Medicine Research Development Fund (RDF160301MMC11).

Author's contributions

MMC conceived the study idea, design and developed the topic guide. SR co-ordinated and supervised the research activities in Pakistan. AA, SS and RA conducted the interviews, transcription, translations and data analysis. MMc co-ordinated and supervised the overall research activities, interpreted and presented the results and wrote the manuscript. NvdB has reviewed the results and contributed to the manuscript. All authors have read, edited and approved the final manuscript for submission.

Acknowledgements

The authors would like to thank Professor Pratima Mittal, Professor Jyotsna Suri and Dr Mansi for their support with ethics applications and data collection in India. A huge thank you to all the women who participated in this study.

For peer review only - http://bmjopen.bmj.com/site/about/guidelines.xhtml

References

1. United Nations. Global Strategy for Women's, Children's and Adolescents' Health, 2016-2030. New York: United Nations; 2015. http://www.who.int/life-course/partners/global-strategy/en/
2. United Nations. Sustainable Development Goals. http://www.un.org/sustainabledevelopment/sustainable-development-goals/
3. World Health Organization. Preamble to the Constitution of the World Health Organization as adopted by the International Health Conference, New York, 19-22 June 1946. http://www.who.int/about/definition/en/print.html
4. McCauley M, Madaj B, White S, Dickinson F, Bar-Zev S, Aminu M, Godia P, Mittal P, Zafar S, van den Broek N. The burden of physical, psychological and social ill-health during and after pregnancy in women in India, Pakistan, Kenya and Malawi. BMJ Glob Health. *In press*.
5. McCauley M, Head J, Lambert J, Zafar S, van den Broek, N. "Keeping family matters behind closed doors": healthcare providers' perceptions and experiences of identifying and managing domestic violence during and after pregnancy. BMC Pregnancy Childbirth. 2017;(17)1:318.
6. Zafar S, Jean-Baptiste R, Rahman A, Neilson JP, van den Broek N. Non-Life Threatening Maternal Morbidity: Cross Sectional Surveys from Malawi and Pakistan. PLoS ONE. 2015;10(9):e0138026.
7. Say L, Souza JP, Pattinson RC, for the WHO working group on Maternal Mortality and Morbidity classifications. Maternal near miss - towards a standard tool for monitoring quality of maternal health care. Best Pract Res Clin Obstet Gynaecol. 2009;23(3):287-96.
8. Chou D, Tunçalp Ö, Firoz T, Barreix M, Filippi V, von Dadelszen P, van den Broek N, Cecatti JG, Say L. Constructing maternal morbidity – towards a standard tool to measure and monitor maternal health beyond mortality. BMC Pregnancy Childbirth. 2016;16:45.
9. Firoz T, Chou D, von Dadelszen P, Agrawal P, Vanderkruik R, Tunçalp O, Magee LA, van den Broek N, Say L. Measuring maternal health: focus on maternal morbidity. Bull World Health Organ. 2013;91(10):794-6.
10. Braun V, Clarke V. Using thematic analysis in psychology. Qual Res Psychol. 2006;3(2):77-101.
11. Equator Network. Consolidated criteria for reporting qualitative research (COREQ): a 32-item checklist for interviews and focus groups. http://www.equator-network.org/reporting-guidelines/coreq/
12. Huber M, Knottnerus JA, Green L, van der Horst H, Jadad AR, Kromhout D, Leonard B, Lorig K, Loureiro MI, van der Meer JW, et al. How should we define health? BMJ. 2011;343:d416
13. World Health Organization. The WHO Application of ICD-10 to deaths during pregnancy, childbirth and the puerperium: ICD-MM. Geneva: World Health Organization; 2012.

For peer review only - http://bmjopen.bmj.com/site/about/guidelines.xhtml

http://www.who.int/reproductivehealth/publications/monitoring/9789241548458/en/

14. Abalos E, Cuesta C, Grosso AL, Chou D, Say L. Global and regional estimates of pre-eclampsia and eclampsia: a systematic review. Eur J Obstet Gynecol Reprod Biol. 2013;170(1):1-7
15. Calvert C, Thomas SL, Ronsmans C, Wagner KS, Adler AJ, Filippi V. Identifying regional variation in the prevalence of postpartum haemorrhage: a systematic review and meta-analysis. PLoS ONE. 2012;7(7):e41114.
16. World Health Organization. Haemoglobin concentrations for the diagnosis of anaemia and assessment of severity. Geneva: World Health Organization; 2011. http://www.who.int/vmnis/indicators/haemoglobin.pdf
17. Velu PP, Gravett CA, Roberts TK, Wagner TA, Zhang JSF, Rubens CE, Gravett MG, Campbell H, Rudan I. Epidemiology and aetiology of maternal bacterial and viral infections in low- and middle-income countries. J Glob Health. 2011;1(2):171-88.
18. World Health Organization. Maternal Mental Health; 2017. http://www.who.int/mental_health/maternal-child/maternal_mental_health/en/
19. Vos T, Barber RM, Bell B, Bertozzi-Villa A, Biryukov S, Bollger I and Global Burden of Disease Study 2013 Collaborators.. Global, regional, and national incidence, prevalence, and years lived with disability for 301 acute and chronic diseases and injuries in 188 countries, 1990-2013: a systematic analysis for the Global Burden of Disease Study 2013. Lancet. 2015;386(9995):743-800.
20. Shrivastava SR, Shrivastava PS, Ramasamy J. Antenatal and postnatal depression: a public health perspective. J Neuros Rural Prac. 2015;6(1):116-9.
21. World Health Organization. Understanding and addressing violence against women. Geneva: World Health Organization; 2012. http://apps.who.int/iris/bitstream/10665/77432/1/WHO_RHR_12.36_eng.pdf
22. World Health Organization. Global and regional estimates of violence against women: prevalence and health effects of intimate partner violence and non-partner sexual violence Geneva: World Health Organization; 2013. http://apps.who.int/iris/bitstream/10665/85239/1/9789241564625_eng.pdf?ua=1
23. World Health Organization. Framing the health workforce agenda for the Sustainable Development Goals: Biennium report 2016-2017 WHO Health Workforce. Geneva: World Health Organization; 2017 http://www.who.int/hrh/BienniumReportRevised2017.pdf
24. Gelaye B, Rondon MB, Araya R, Williams MA. Epidemiology of maternal depression, risk factors, and child outcomes in low-income and middle-income countries. Lancet Psychiatry. 2016;3(10):973.
25. Zakar R, Nasrullah M, Zakar MZ, Ali H. The association of intimate partner violence with unintended pregnancy and pregnancy loss in Pakistan. Int J Gynecol Obstet. 2016;133:26-31.

For peer review only - http://bmjopen.bmj.com/site/about/guidelines.xhtml

26. Satyanarayana VA, Lukose A, Srinivasan K. Maternal mental health in pregnancy and child behavior. Indian J Psychiatry. 2011;53(4):351.
27. National Institute for Health and Care Excellence. Antenatal and postnatal mental health: clinical management and service guidance. London: NICE; 2015. https://www.nice.org.uk/guidance/cg192
28. World Health Organization. WHO recommendations on antenatal care for a positive pregnancy experience. Geneva: World Health Organization; 2016. http://www.who.int/reproductivehealth/publications/maternal_perinatal_health/anc-positive-pregnancy-experience/en/
29. Gureje O, Oladeji BD, Araya R, Montgomery AA, Kola L, Kirmayer L, Zelkowitz P, Groleau D, Kimayer L, Zelkowitz P, Groleau D. Expanding care for perinatal women with depression (EXPONATE): Study protocol for a randomized controlled trial of an intervention package for perinatal depression in primary care. BMC Psychiatry. 2015;15(1):136.

For peer review only

For peer review only - http://bmjopen.bmj.com/site/about/guidelines.xhtml

COREQ (COnsolidated criteria for REporting Qualitative research) Checklist

A checklist of items that should be included in reports of qualitative research. You must report the page number in your manuscript where you consider each of the items listed in this checklist. If you have not included this information, either revise your manuscript accordingly before submitting or note N/A.

Topic	Item No.	Guide Questions/Description	Reported on Page No.
**Domain 1: Research team and reflexivity**			
Personal characteristics			
Interviewer/facilitator	1	Which author/s conducted the interview or focus group?	
Credentials	2	What were the researcher's credentials? E.g. PhD, MD	
Occupation	3	What was their occupation at the time of the study?	
Gender	4	Was the researcher male or female?	
Experience and training	5	What experience or training did the researcher have?	
Relationship with participants			
Relationship established	6	Was a relationship established prior to study commencement?	
Participant knowledge of the interviewer	7	What did the participants know about the researcher? e.g. personal goals, reasons for doing the research	
Interviewer characteristics	8	What characteristics were reported about the inter viewer/facilitator? e.g. Bias, assumptions, reasons and interests in the research topic	
**Domain 2: Study design**			
Theoretical framework			
Methodological orientation and Theory	9	What methodological orientation was stated to underpin the study? e.g. grounded theory, discourse analysis, ethnography, phenomenology, content analysis	
Participant selection			
Sampling	10	How were participants selected? e.g. purposive, convenience, consecutive, snowball	
Method of approach	11	How were participants approached? e.g. face-to-face, telephone, mail, email	
Sample size	12	How many participants were in the study?	
Non-participation	13	How many people refused to participate or dropped out? Reasons?	
Setting			
Setting of data collection	14	Where was the data collected? e.g. home, clinic, workplace	
Presence of non-participants	15	Was anyone else present besides the participants and researchers?	
Description of sample	16	What are the important characteristics of the sample? e.g. demographic data, date	
Data collection			
Interview guide	17	Were questions, prompts, guides provided by the authors? Was it pilot tested?	
Repeat interviews	18	Were repeat inter views carried out? If yes, how many?	
Audio/visual recording	19	Did the research use audio or visual recording to collect the data?	
Field notes	20	Were field notes made during and/or after the inter view or focus group?	
Duration	21	What was the duration of the inter views or focus group?	
Data saturation	22	Was data saturation discussed?	
Transcripts returned	23	Were transcripts returned to participants for comment and/or	

For peer review only

Topic	Item No.	Guide Questions/Description	Reported on Page No.
		correction?	
**Domain 3: analysis and findings**			
Data analysis			
Number of data coders	24	How many data coders coded the data?	
Description of the coding tree	25	Did authors provide a description of the coding tree?	
Derivation of themes	26	Were themes identified in advance or derived from the data?	
Software	27	What software, if applicable, was used to manage the data?	
Participant checking	28	Did participants provide feedback on the findings?	
Reporting			
Quotations presented	29	Were participant quotations presented to illustrate the themes/findings? Was each quotation identified? e.g. participant number	
Data and findings consistent	30	Was there consistency between the data presented and the findings?	
Clarity of major themes	31	Were major themes clearly presented in the findings?	
Clarity of minor themes	32	Is there a description of diverse cases or discussion of minor themes?	

Developed from: Tong A, Sainsbury P, Craig J. Consolidated criteria for reporting qualitative research (COREQ): a 32-item checklist for interviews and focus groups. *International Journal for Quality in Health Care*. 2007. Volume 19, Number 6: pp. 349 – 357

Once you have completed this checklist, please save a copy and upload it as part of your submission. DO NOT include this checklist as part of the main manuscript document. It must be uploaded as a separate file.

For peer review only

BMJ Open

"Good health means being mentally, socially, emotionally and physically fit": Women's understanding of health and ill-health during and after pregnancy in India and Pakistan: a qualitative study

Journal:	*BMJ Open*
Manuscript ID	bmjopen-2018-028760.R1
Article Type:	Research
Date Submitted by the Author:	28-May-2019
Complete List of Authors:	McCauley, Mary; Liverpool School of Tropical Medicine, Centre for Maternal and Newborn Health<br>Avais, Ayesha ; Child Advocacy International Pakistan<br>Agrawal, Ritu; Vardhman Mahavir Medical College and Safdarjung Hospital<br>Saleem, Shumaila; Child Advocacy International Pakistan<br>Zafar, S; Fazaia Medical College<br>van den Broek, Nynke; Liverpool School of Tropical Medicine, Centre for Maternal and Newborn Health
**Primary Subject Heading**:	Obstetrics and gynaecology
Secondary Subject Heading:	Evidence based practice, Global health
Keywords:	Maternal medicine < OBSTETRICS, Antenatal care, Postnatal care, Maternal morbidity, Understanding of health and ill-health

SCHOLARONE™
Manuscripts

"Good health means being mentally, socially, emotionally and physically fit": Women's understanding of health and ill-health during and after pregnancy in India and Pakistan: a qualitative study

Authors:

MCCAULEY, Mary[1]*

AVAIS, Ayesha Rasheeda[2]

AGRAWAL, Ritu [3]

SALEEM, Shumaila[2]

ZAFAR, Shamsa[4]

VAN DEN BROEK, Nynke[1]

[1]Centre for Maternal and Newborn Health, Liverpool School of Tropical Medicine, United Kingdom

[2]Child Advocacy International Pakistan, Islamabad, Pakistan

[3]Safdarjung Hospital, New Delhi, India

[4]Fazaia-Medical College, Air University, Islamabad, Pakistan

*Corresponding author: mary.mccauley@lstmed.ac.uk

Word count: 3464

Ethical Approval: The Liverpool School of Tropical Medicine, Liverpool, United Kingdom, Safdarjung Hospital, New Delhi and the National Bioethics Committee, Islamabad, Pakistan all granted ethical approval.

Disclosure of Funding Received: This study was funded by a Research Development Fund (RDF160301MMC11) from the Liverpool School of Tropical Medicine, Liverpool, United Kingdom.

Précis (Max 25 words)

Women in India and Pakistan recognise that ill-health is not just related to physical disease. Care packages need to be modified to address psycho-social maternal morbidity.

Short Title:

Women's views on health and ill-health during and after pregnancy in India and Pakistan.

Keywords: Health, ill-health, antenatal care, postnatal care, pregnancy, maternal morbidity.

For peer review only - http://bmjopen.bmj.com/site/about/guidelines.xhtml

For peer review only

Abstract

Objective

To explore what women, consider health and ill-health to be, in general, and during and after pregnancy. Women's views on how to approach and discuss all components of ill-health as part of routine screening during and after pregnancy, including psycho-social morbidity were also explored.

Setting

A public hospital in New Delhi, India and a public hospital in Islamabad, Pakistan.

Participants

130 women attending for routine antenatal or postnatal care at the study healthcare facilities.

Interventions

Data collection was conducted using focus group discussions and key informant interviews. Transcribed interviews coded by topic and grouped into categories. Thematic framework analysis was undertaken to identify emerging themes.

Results

Most women are aware that health is a multidimensional concept, not simply physical symptoms or identified medical disease. Concepts of "good health" included a nutritious diet; having an ideal weight; absence of disease; and a supportive family environment. Ill-health consisted of physical symptoms and disease; stress and tension; and domestic violence and alcohol use in the family. Women are aware that the health or ill-health of the mother and baby are interlinked. In addition to a full physical assessment, most women welcome psycho-social ill-health enquiries. Reported barriers to routine enquiry regarding psycho-social aspects of maternal health included women's perceptions that these issues are "personal"; that healthcare provider's do not have the time and/or cannot provide further care, even if psycho-social morbidity is disclosed.

Conclusions

Women have a good understanding of the comprehensive nature of health and ill-health during and after pregnancy. Women report that inquiry regarding psycho-social ill-health is currently not part of routine maternity care, but most welcome such an assessment.

For peer review only - http://bmjopen.bmj.com/site/about/guidelines.xhtml

Keywords

Health, ill-health, maternal health, women, antenatal care, postnatal care, qualitative research, perceptions.

Article summary

Strengths and limitations of this study

• Maternal ill-health is an international public health concern.

• This is the first study to obtain women's own opinions on what they consider health and ill-health to be during and after pregnancy.

• A large number of women (n=130) attending for routine antenatal or postnatal care were interviewed in urban settings in India and Pakistan.

• Our findings indicate that in addition to a full physical review, most women would welcome a psycho-social assessment as part of routine care.

For peer review only

For peer review only - http://bmjopen.bmj.com/site/about/guidelines.xhtml

Introduction

All women have the right to the highest attainable standard of health and well-being including physical, psychological and social aspects [1]. The Sustainable Development Goals (SDG) developed by the United Nations and endorsed by 193 countries worldwide, envision that that every woman in every setting has an equal chance to survive and thrive [2]. SDG three is to 'ensure healthy lives and promote well-being for all' [2]. The Global Strategy strives for a world in which every mother can enjoy a wanted and healthy pregnancy and childbirth and that pregnancy is an opportunity for a woman to receive care to "thrive" [1]. Currently this is not the case in many low-and middle- income countries (LMIC). As part of the need to develop innovative strategies to achieve SDG 3, there has been a renewed focus on maternal ill-health or "maternal morbidity" in terms of definition, measurement, and interventions [3-10]. The burden of disease of the various aspects of health (physical, psychological and social) during and after pregnancy and their impact on women and/or pregnancy outcomes are currently not well documented in a comprehensive, standardised approach [3,4]. It is suggested that although maternal ill-health occurs across all countries, cultures, religions, socioeconomic status, and ages, the burden of maternal morbidity is expected to be higher in LMIC with an estimated three out of four women suffering at least one form of maternal morbidity during or after their pregnancy [3,4].

Maternal ill-health or morbidity is a complex and broad concept and can be understood and described in various ways depending on different perspectives [8-10]. For example, women, men, families, communities, healthcare providers and policy makers may have mixed views and perceptions on what maternal ill-health is and the effect or risk of maternal morbidity during and after pregnancy for the women and/or her baby [9]. Maternal morbidity has been defined as "any health condition that is attributed to or aggravated by pregnancy and childbirth which has a negative impact on the woman's wellbeing" [8]. However, there is still a lack of understanding and lack of data regarding what women themselves consider ill-health to be (physical and/or psychological and/or social) and their experiences (subjective measures) compared to clinical findings (objective measures) when assessed and defined as "morbidity" by a healthcare provider [3-10]. The WHO have recently highlighted the need for qualitative studies that explore women's lived experiences of ill-health and perceptions of

For peer review only - http://bmjopen.bmj.com/site/about/guidelines.xhtml

their health and health needs during and after pregnancy [9-11]. This study therefore sought to explore what women attending for routine antenatal care (ANC) or postnatal care (PNC) at healthcare facilities in India and Pakistan consider health and ill-health to be in general, and, what they consider health and ill-health to be, during and after pregnancy. As a component of maternal ill-health, women's views on how best to approach psycho-social morbidity, were assessed.

Materials

Study design and setting

Experienced female researchers (AA, RA, SS) interviewed a total of 130 women. Data collection was conducted using focus group discussions (FGD) to interview 60 women attending for routine ANC or PNC in the outpatient clinics at a large public hospital in New Delhi, India. FDGs and key informant interviews (KII) were used to interview 70 women attending for routine ANC or PNC in the outpatient clinics at a large public hospital in Islamabad, Pakistan. The sampling frame included (1) women who were pregnant or who have given birth within the past six weeks and (2) women who above the legal age of consent. Purposive sampling was used to approach women attending each study setting healthcare facility for routine ANC or PNC care. Women were only included once in the study. All interviews and FGDs were held in quiet locations away from the clinical areas at the relevant hospitals.

Participants and settings

All women attending for routine ANC or PNC in the study healthcare facilities were invited to participate. The study settings were two large public urban hospitals serving women with low-lower middle socio-economic status; and with varied education levels [4]. Similar proportions of women were included from four different stages of pregnancy and FGDs included women within the same assessment stage of pregnancy (first half of pregnancy (≤20 weeks); second half of pregnancy (>20 weeks); early postnatal stage (day 1-7); or late postnatal stage (week 2-6). Women were purposively selected and recruited sequentially until the number of anticipated FGD and key informant interviews was reached, and saturation was achieved.

For peer review only - http://bmjopen.bmj.com/site/about/guidelines.xhtml

Patient and Public Involvement

All women recruited to this study were patients attending for routine ANC or PNC in the study healthcare facilities. The topic guide was piloted among several patients in each study setting and amendments made prior to the start of data collection. The main results of this study have been verbally disseminated to patient representatives in each study setting.

Topic guide

A topic guide was developed to guide the interviews and was piloted in the United Kingdom with clinical researchers from LMIC (Supplementary document 1). The topic guide was then translated into the local study setting language, back-translation was conducted, and the topic guide was further piloted in each study setting. As a result, the topic guide was refined; for example, some wording was changed in the local language and the introduction was amended to ensure that the women were aware that we sought to assess their general views and not their own personal experiences (if any) of different types of ill-health. The topic guide served as a flexible tool to facilitate the facilitators in obtaining the women's opinions whilst ensuring that the interview remained on topic. The topic guide also acted as a cue to ask more probing questions to further understand women' beliefs and perceptions. The topic guide included three main subject areas:

1) perception and understanding of health and ill-health in general;

2) perception and understanding of health and ill-health during and after pregnancy;

3) views on enquiry regarding psycho-social morbidity during and after pregnancy; and suggestions on how best to approach all components of health.

For peer review only

Data Collection

Prior to interview, all eligible women were approached and given verbal and written information regarding the study including a brief overview of the research aims and interview questions. Appointments for FGD or key informant interviews were then scheduled for a later time convenient for the women. The FGD and key informant interviews were conducted in the local language (India-Hindi and Pakistan-Urdu) by the local female trained and experienced researchers (AA, RA, SS). On average the FGDs lasted 60-90 minutes, the KIIs lasted 30 minutes. All FGD and key informant interviews were conducted face-to-face,

For peer review only - http://bmjopen.bmj.com/site/about/guidelines.xhtml

recorded on a digital recording device and transcribed in the local language upon completion by the local researchers (AA, RA, SS). All efforts were made to emphasise confidentiality for interviews to ensure women were confident in giving honest answers and member checking was used to assure correct interpretation. Interviewing women from different stages of pregnancy, different backgrounds, and different countries broadened the scope of the topic and enabled transferability to be achieved to an extent.

Data analysis

In each study setting, transcribed interviews were translated into English and initially manually open coded following data familiarisation (reading and re-reading of the transcripts several times) by the local researchers (RA in India; AA and SS in Pakistan). In a sub-sample of cases, and prior to translation, participants were asked to review the coded transcripts to verify the accuracy and appropriateness of the applied code. Using thematic analysis, codes were identified from the transcripts and grouped into categories independently by the local researchers, enabling the first abstraction of data in each setting [11]. Thematic framework analysis of the categories was then undertaken. The separate results were then brought together and refined to agree on the key themes [11]. All of the coded data was reviewed by other members of the research team (MMc in India and SZ in Pakistan) for sense checking. In addition, all members of the research team reviewed, and critiqued emerging themes and sub-themes and any discrepancies were discussed, and consensus agreed. The reporting of the results adheres to the COREQ guidelines [12].

Ethics

The Liverpool School of Tropical Medicine, Liverpool, United Kingdom, granted full ethical approval (LSTM 16-054RS). Ethical approval was also obtained from the Research and Ethics Committee, Vardhman Mahavir Medical College and Safdarjung Hospital, New Delhi, India (ICE/SJH/VMMC/Project/October-2016/688); and the National Bioethics Committee, Islamabad, Pakistan (No.4-87/16/NBC-159-Addendum/RDC/3634). Written informed consent was obtained from each woman who participated in the study.

For peer review only - http://bmjopen.bmj.com/site/about/guidelines.xhtml

Results

A total of 130 women were interviewed (Table 1). Three women invited to participate declined due to time constraints. A total of 83 women were pregnant at the time of interview and 47 had given birth with the past six weeks. Most women were either in their first pregnancy or had one previous pregnancy. A minority of women had more than two previous pregnancies. The age range was from 20-36 with a mean of 26 years old. There were no distinct differences in emerging themes or sub-themes from women from India or Pakistan. Therefore, all quotes illustrating the results are combined for both countries and displayed in tables and linked to the narrative text.

What is health?

Women perceived "good health" in general as having a good diet; having an ideal weight; absence of physical disease; absence of stress and tension; and a supportive family environment (Table 2). Most women expressed that the ability to have a good diet would result in good health (Table 2, Q1-Q3). Weight was mentioned in India and Pakistan, however, some women's perception of this differed (Table 2, Q4-Q5). In both settings, women reported that health meant lack of disease and good energy levels (Table 2, Q6-Q7). Most women reported that health was not just physical health but includes mental health (Table 2, Q8-Q10). Most women highlighted that a supportive environment with family were important for good health (Table 2, Q11-Q13).

What is ill-health?

The emerging themes related to perceived ill-health included; physical symptoms and disease; tension; and an abusive and stressful home environment. Many women expressed that ill-health was "pain, flu, fever, sore throat, weakness, body ache, headache, and swelling" (Table 2, Q14-Q17). Ill-health was also described in terms of disease, for example HIV/AIDS (Table 2, Q18). Mental ill-health was often considered a health concern and stress and tension was reported by many women as a component of or factor leading to mental ill-health (Table 2, Q19-Q20).

For peer review only - http://bmjopen.bmj.com/site/about/guidelines.xhtml

What is health during and after pregnancy?

During and after pregnancy a nutritious diet, medication, rest, extra care, and a peaceful home environment were mentioned as important. Most women reported that diet is even more important when a woman is pregnant (Table 3, Q21-Q22). Many women mentioned that pregnant women needed medication and more care from the family and healthcare providers (Table 3, Q23-Q26). Many women understood that their health was "lack of tension" and that their (or ill-health) was linked to that of their baby (Table 3, Q26-Q28).

What is ill-health during and after pregnancy?

Perceived ill-health during and after pregnancy included the perception that pregnancy is a "disease", and that there was "no health in pregnancy", as all "the energy goes to the baby and the mother becomes weaker". Women also considered some physical symptoms and discomfort as "normal" during pregnancy. This included nausea and vomiting, back pain and difficulty sleeping. However, many women reported that ill-health during and after pregnancy was "vomiting, high blood pressure, bleeding, swelling, abdominal pain" (Table 3, Q29). Many women were aware that these symptoms could be dangerous (Table 3, Q30-Q31). Stress and tension were considered important components of ill-health by most women (Table 3, Q32). An abusive family environment was highlighted as examples of social ill-health in women during and after pregnancy (Table 3, Q33-Q35). There was some discussion regarding gender preference and it was suggested by a minority of women that the gender of a baby created stress and tension for women (Table 3, Q36-Q37).

Should healthcare providers ask about psycho-social ill-health?

An emerging theme regarding what women consider health and ill-health to be, was the importance of psycho-social well-being. Women described psychological ill-health such as "depression", "weeping", stress", "tension", "grief", "loss of appetite" and "unhappiness". Women reported social concerns such as "fights in the home", "domestic violence", "poor family environment", "quarrels and verbal abuse", "family or husband alcohol misuse", and an "expectation of a male child".

For peer review only - http://bmjopen.bmj.com/site/about/guidelines.xhtml

Women were therefore asked questions regarding how best to approach and address the concept of psycho-social ill-health for women during and after pregnancy. Most women reported that healthcare providers ask pregnant women questions regarding physical ill-health only (Table 4, Q38-Q40). Most women felt that there was a lack of enquiry from healthcare providers regarding psycho-social ill-health during and after pregnancy. Most women felt that healthcare providers could and should ask women about their psycho-social health (Table 4, Q41-Q43). Many women said that it would be beneficial to speak to their healthcare provider if they were feeling “stressed” and “under tension” as the healthcare provider might be able to provide support and advice. However, a few women explained these types of questions might be considered unacceptable, because of lack of education and understanding among some women (Table 4, Q44-Q45). There was some concern that some women would prefer not to talk about their psycho-social ill-health as they may feel afraid, ashamed or uncomfortable (Table 4, Q46-Q48). A small minority of women in Pakistan felt it was not the role of the healthcare provider to ask questions regarding psycho-social health, as this was their “personal life”. Women discussed the repercussions from husbands and family if domestic violence during and after pregnancy would be disclosed by a woman to a healthcare provider (Table 4, Q49-Q51). There were concerns that a woman would not be allowed to access care again. There was a lot of discussion about this with sometimes conflicting opinions; ranging from it not being right for a woman to disclose domestic violence, or psychological ill-health such as stress and tension; to a consideration that healthcare providers should ask and would provide help. A few women said that if they themselves were asked questions regarding depression or domestic violence, they would seek care from another healthcare provider to avoid these questions. Many women recognised that there were existing barriers for healthcare providers which made it difficult for them to ask women questions regarding psycho-social ill-health during and after pregnancy (Table 4, Q52-Q53). Such barriers included the perceived heavy workload of healthcare providers and lack of time to explore psycho-social concerns (Table 4, Q51). Other women explained that “they can ask, but what can they do?” and that “nothing can be done” to help. Some women did offer suggestions of how healthcare providers could best ask questions regarding psycho-social ill-health during and after pregnancy. A more empathic approach to women were suggested including politeness and friendliness were suggested. Confidentiality was

For peer review only - http://bmjopen.bmj.com/site/about/guidelines.xhtml

highlighted as important by many women. Some women suggested that healthcare providers need not go into such detailed questions but keep any questions simple (Table 4, Q54-Q55).

Discussion

Statement of principal findings

Most women who took part in this study had clear opinions and had a good understanding that health includes more than physical components of health and that the health of the mother was linked to that of the baby. Good diet, healthy weight, rest and a supportive home environment contributed to good health; whereas physical disease symptoms, complications of pregnancy, as well as stress, tension and an abusive family environment contributed to ill-health. Women were aware of physical common discomforts and of more severe complications and dangers signs, as ill-health related to pregnancy. Women considered psycho-social ill-health as important but reported that healthcare providers do not currently ask women how they are feeling or ask after their psycho-social health. However, most women would be happy if healthcare providers did so. A minority of women considered psycho-social concerns as "personal" and reported that there may be barriers to asking such questions.

Strengths of the study

To the best of our knowledge, this is the first study from two LMIC settings (India and Pakistan) to obtain women's own opinions on what they might consider health and ill-health to be in general and during and after pregnancy. A range of women from four different stages of pregnancy were interviewed resulting in a wide spectrum of responses. Most women approached welcomed the discussion surrounding health, and ill-health during and after pregnancy, and were keen to contribute to solutions in their settings regarding healthcare providers roles in routine enquiry regarding other components of health including psycho-social aspects, and not just physical health.

Limitations of the study

This study population included women attending for routine ANC or PNC in public secondary level healthcare facilities in urban settings and excludes other women who may have

For peer review only - http://bmjopen.bmj.com/site/about/guidelines.xhtml

alternative perspectives or different insights. There is a need to assess the views of women in other LMIC and including community and rural settings, as these women may have different perceptions and understandings.

How does this study relate to other literature?

There is lack of literature regarding the understanding of what women consider health and ill-health to be during and after pregnancy in India and Pakistan. There is also debate that the current definition of health [13] is out-dated and needs to be re-formulated to consider health in a context of functionality, capacity, adaptability and the ability to perform activities of daily living despite having a disease or disability [14]. There have been various proposals to develop the definition of health using the concept of "health, as the ability to adapt and to self-manage" but with a continued emphasis on the importance of the three domains of health: physical, psychological, and social [14]. Aspects of physical ill-health in women during and after pregnancy in LMIC settings are well documented and there are many recommendations and interventions developed to diagnose and manage these conditions; for example, anaemia, haemorrhage, hypertensive disorders of pregnancy, infection, and complications of obstructed labour [15-19]. With regards to psycho-social ill-health in women during and after pregnancy in LMIC, there is data to demonstrate that depression and domestic violence represent a large burden of ill-health and have a negative impact on a woman's health and well-being [20-24]. However, to date, clinical practice in low resource settings rarely takes psycho-social ill-health into account when assessing a woman's health needs during and after pregnancy [24]. However, this study demonstrates that most women would welcome such an assessment but report that their healthcare providers do not current ask; and even if they did, some women report that "nothing that can be done". This may be due to a shortage of human resources but also because of healthcare providers' perceptions that these issues are not "ill-health" per se and because of perceived cultural sensitivities related to psycho-social issues [3,4, 24-26].

It is well recognised that maternal physical and psycho-social ill-health is linked to newborn babies' health and women in our study demonstrated an understanding that all aspects of ill-health are associated with poor fetal and newborn health outcomes [27-30]. The detrimental

For peer review only - http://bmjopen.bmj.com/site/about/guidelines.xhtml

impact of psycho-social morbidity on the overall health and well-being of mothers (and their babies) during and after pregnancy has resulted in public health policy in high income countries such as the United Kingdom (UK), where enquiry regarding of psycho-social ill-health including depression and domestic violence, is routinely conducted during and after pregnancy [31]. The WHO have produced clinical and policy guidelines on how to comprehensively care for women during and after pregnancy including identification, safety assessment and planning, communication and clinical skills, documentation and provision of referral pathways for psycho-social ill-health [32]. However, the feasibility of implementation and acceptability of this guidance in countries, such as India and Pakistan are currently uncertain. There is debate as to the cadre of healthcare providers most suitable to undertake routine enquiry regarding for and/or manage psycho-social ill-health in LMIC [5,25,33]. In many high-income countries, specially trained midwives routinely assess, screen, support and provide further referral for women with psycho-social morbidity [31]. This study shows that most women are keen to be asked questions regarding their psycho-social well-being during and after pregnancy, as part of routine maternity care. However, a minority of women do not wish to be asked.

Currently, there is good coverage and uptake of antenatal care, but content needs to be adapted to ensure good quality comprehensive care that is respectful, integrated and delivers physical, psycho-social care, with agreed country specific additional content. Although, psycho-social ill-health is clearly a real matter of concern for women, this study was not designed to explore this in further detail. Further research is needed to explain in more depth what these problems are, and who and how they can be addressed. It is important that healthcare providers are supported to provide good quality care for women and that this goes beyond simply the physical aspects of health and is inclusive of psycho-social well-being.

Conclusion

Women reported that health and ill-health is not just physical but also includes psychological and social components. Maternal ill-health is common among women attending for antenatal and postnatal care [4]. Women are increasingly accessing care during and after pregnancy and there is a window of opportunity to adapt and amend available care packages to include

For peer review only - http://bmjopen.bmj.com/site/about/guidelines.xhtml

comprehensive enquiry regarding and, where needed, support for psycho-social ill-health [3, 10]. This study demonstrates that most women are keen to be asked questions regarding their psycho-social well-being and provides simple suggestions that healthcare providers can use to improve the enquiry regarding psycho-social ill-health among women during and after pregnancy. There is a need for further research to help develop interventions and deliver maternity care in a way that meets the health needs of women in a comprehensive and holistic manner, including psycho-social components [3, 8-10].

Abbreviations

ANC: antenatal, **FGD**: Focus group discussion, **KII**: Key informant interviews, **LMIC**: Low and middle-income country, **LSTM**: Liverpool School of Tropical Medicine, **SDG**: Sustainable Development Goals, **PNC:** postnatal, **UK**: United Kingdom, **WHO**: World Health Organization

Declarations

Ethics approval and consent to participate

The Liverpool School of Tropical Medicine, Liverpool, United Kingdom, granted full ethical approval (LSTM 16-054RS). Ethical approval was also obtained from the Research and Ethics Committee, Vardhman Mahavir Medical College and Safdarjung Hospital, New Delhi, India (ICE/SJH/VMMC/Project/October-2016/688); and the National Bioethics Committee, Islamabad, Pakistan (No.4-87/16/NBC-159-Addendum/RDC/3634). Written informed consent was obtained from each woman who participated in the study.

Consent for publication

Written informed consent was obtained from all women in the study, including audio-recording and the use of excerpts in publications and reports.

Availability of data and material

The datasets used and analysed during the current study are available from the corresponding author on reasonable request.

Competing interests

The authors declare that they have no competing interests.

For peer review only - http://bmjopen.bmj.com/site/about/guidelines.xhtml

Funding

Disclosure of funding received: This study was funded by the Liverpool School of Tropical Medicine Research Development Fund (RDF160301MMC11).

Author's contributions

MMC conceived the study idea, design and developed the topic guide. SZ co-ordinated and supervised the research activities in Pakistan. AA, SS and RA conducted the interviews, transcription, translations and data analysis. MMc co-ordinated and supervised the overall research activities, interpreted and presented the results and wrote the manuscript. NvdB has reviewed the data, interpreted the results and contributed to the manuscript. All authors have read, edited and approved the final manuscript for submission.

Acknowledgements

The authors would like to thank Professor Pratima Mittal, Professor Jyotsna Suri and Dr Mansi for their support with ethics applications and data collection in India. A huge thank you to all the women who participated in this study.

For peer review only - http://bmjopen.bmj.com/site/about/guidelines.xhtml

References

1. United Nations. Global Strategy for Women's, Children's and Adolescents' Health, 2016-2030. New York: United Nations; 2015. http://www.who.int/life-course/partners/global-strategy/en/
2. United Nations. Sustainable Development Goals. http://www.un.org/sustainabledevelopment/sustainable-development-goals/
3. McCauley M, Madaj B, White SA, *et al*. Burden of physical, psychological and social ill-health during and after pregnancy among women in India, Pakistan, Kenya and Malawi. BMJ Glob Health 2018;**3**:e000625.
4. Zafar S, Jean-Baptiste R, Rahman A, *et al*. Non-Life Threatening Maternal Morbidity: Cross Sectional Surveys from Malawi and Pakistan. PLoS ONE 2015;**10**:e0138026.
5. Say L, Souza JP, Pattinson RC, for the WHO working group on Maternal Mortality and Morbidity classifications. Maternal near miss - towards a standard tool for monitoring quality of maternal health care. Best Pract Res Clin Obstet Gynaecol 2009;**23**:287-96.
6. Chou D, Tunçalp Ö, Firoz T, *et al*. Constructing maternal morbidity – towards a standard tool to measure and monitor maternal health beyond mortality. BMC Pregnancy Childbirth 2016;**16**:45.
7. Barreix M, Barbour K, McCaw-Binns A, *et al*. Standardizing the measurement of maternal morbidity: Pilot study results. Int J Gynecol Obstet 2018;**141**:10-9.
8. Say L, Chou D. Maternal morbidity: Time for reflection, recognition, and action. Int J Gynecol Obstet 2018;**141**:1-3.
9. Filippi V, Chou D, Barreix M, Say L. A new conceptual framework for maternal morbidity. Int J Gynecol Obstet 2018;**141**:4-9.
10. Firoz T, Chou D, von Dadelszen P, *et al*. Measuring maternal health: focus on maternal morbidity. Bull World Health Organ 2013;**91**:794-6.
11. Braun V, Clarke V. Using thematic analysis in psychology. Qual Res Psychol 2006;**3**:77-101.
12. Equator Network. Consolidated criteria for reporting qualitative research (COREQ): a 32-item checklist for interviews and focus groups. http://www.equator-network.org/reporting-guidelines/coreq/
13. World Health Organization. Preamble to the Constitution of the World Health Organization as adopted by the International Health Conference, New York, 19-22 June 1946. http://www.who.int/about/definition/en/print.html
14. Huber M, Knottnerus JA, Green L, *et al*. How should we define health? BMJ 2011;**343**:d416
15. World Health Organization. The WHO Application of ICD-10 to deaths during pregnancy, childbirth and the puerperium: ICD-MM. Geneva: World Health Organization; 2012. http://www.who.int/reproductivehealth/publications/monitoring/9789241548458/en/

For peer review only - http://bmjopen.bmj.com/site/about/guidelines.xhtml

16. Abalos E, Cuesta C, Grosso AL, *et al*. Global and regional estimates of pre-eclampsia and eclampsia: a systematic review. Eur J Obstet Gynecol Reprod Biol 2013;**170**:1-7
17. Calvert C, Thomas SL, Ronsmans C, *et al*. Identifying regional variation in the prevalence of postpartum haemorrhage: a systematic review and meta-analysis. PLoS ONE 2012;**7**:e41114.
18. World Health Organization. Haemoglobin concentrations for the diagnosis of anaemia and assessment of severity. Geneva: World Health Organization; 2011. http://www.who.int/vmnis/indicators/haemoglobin.pdf
19. Velu PP, Gravett CA, Roberts TK, *et al*. Epidemiology and aetiology of maternal bacterial and viral infections in low- and middle-income countries. J Glob Health 2011;**1**:171-88.
20. World Health Organization. Maternal Mental Health. Geneva: World Health Organization; 2017. http://www.who.int/mental_health/maternal-child/maternal_mental_health/en/
21. Vos T, Barber RM, Bell B, *et al*. Global, regional, and national incidence, prevalence, and years lived with disability for 301 acute and chronic diseases and injuries in 188 countries, 1990-2013: a systematic analysis for the Global Burden of Disease Study 2013. Lancet 2015;**386**:743-800.
22. Shrivastava SR, Shrivastava PS, Ramasamy J. Antenatal and postnatal depression: a public health perspective. J Neuros Rural Prac 2015;**6**:116-9.
23. McCauley M, Brown A, Ofosu B, van den Broek N. "I just wish it becomes part of routine care?": Healthcare providers' knowledge, attitudes and perceptions of screening for maternal mental health during and after pregnancy. BMC Psychiatry (in press)
24. McCauley M, Head J, Lambert J, et al. "Keeping family matters behind closed doors": healthcare providers' perceptions and experiences of identifying and managing domestic violence during and after pregnancy. BMC Pregnancy Childbirth 2017;17:318.
25. World Health Organization. Understanding and addressing violence against women. Geneva: World Health Organization; 2012. http://apps.who.int/iris/bitstream/10665/77432/1/WHO_RHR_12.36_eng.pdf
26. World Health Organization. Global and regional estimates of violence against women: prevalence and health effects of intimate partner violence and non-partner sexual violence Geneva: World Health Organization; 2013. http://apps.who.int/iris/bitstream/10665/85239/1/9789241564625_eng.pdf?ua=1
27. World Health Organization. Framing the health workforce agenda for the Sustainable Development Goals: Biennium report 2016-2017 WHO Health Workforce. Geneva: World Health Organization; 2017 http://www.who.int/hrh/BienniumReportRevised2017.pdf
28. Gelaye B, Rondon MB, Araya R, Williams MA. Epidemiology of maternal depression, risk factors, and child outcomes in low-income and middle-income countries. Lancet Psychiatry 2016;**3**:973.

For peer review only

For peer review only - http://bmjopen.bmj.com/site/about/guidelines.xhtml

29. Zakar R, Nasrullah M, Zakar MZ, Ali H. The association of intimate partner violence with unintended pregnancy and pregnancy loss in Pakistan. Int J Gynecol Obstet 2016;**133**:26-31.
30. Satyanarayana VA, Lukose A, Srinivasan K. Maternal mental health in pregnancy and child behavior. Indian J Psychiatry 2011;**53**:351.
31. National Institute for Health and Care Excellence. Antenatal and postnatal mental health: clinical management and service guidance. London: NICE; 2015. https://www.nice.org.uk/guidance/cg192
32. World Health Organization. WHO recommendations on antenatal care for a positive pregnancy experience. Geneva: World Health Organization; 2016. http://www.who.int/reproductivehealth/publications/maternal_perinatal_health/anc-positive-pregnancy-experience/en/
33. Gureje O, Oladeji BD, Araya R, *et al*. Expanding care for perinatal women with depression (EXPONATE): Study protocol for a randomized controlled trial of an intervention package for perinatal depression in primary care. BMC Psychiatry 2015;**15**:136.

For peer review only

Table 1. Participants

Stage of pregnancy	India		Pakistan		Total number of women
	Method	Number of women	Method	Number of women	
First half of pregnancy (≤20 weeks)	2 FGD	16	2 FGD	26	42
Second half of pregnancy (>20 weeks)	3 FGD	17	2 FGD	24	41
Early postnatal stage (day 1-7)	3 FGD	22	10 KII	10	32
Late postnatal stage (week 2-6)	1 FGD	5	10 KII	10	15
**TOTAL**		**60**		**70**	**130**

For peer review only

For peer review only - http://bmjopen.bmj.com/site/about/guidelines.xhtml

Table 2: Womens' understanding of health and ill-health in general

Health Sub-theme	Illustrative quotes	
Diet	Q1	"If a person is happy but not eating well, how can she be healthy? Diet is so important." (FGD 1, ANC, Pakistan)
	Q2	"Good health is good food." (KII 6, PNC, Pakistan)
	Q3	"Health means to have balanced diet." (FGD 5, ANC, India)
Weight	Q4	"One should be strong and a bit bulky." (FGD 1, ANC, India)
	Q5	"Healthy women are those who are slim and have strength." (KII 3, PNC, Pakistan)
Energy	Q6	"Good health is when there is no disease." (FGD 3, ANC, Pakistan)
	Q7	"Women should have the energy to do the chores, that means they are healthy." (FGD 4, ANC, Pakistan)
Psychological components of health	Q8	"Being healthy means having no stress or worries and having a tension-free life." (FGD 5, ANC, India)
	Q9	"There is health of the mind and other body parts. If one body part is missing or is not healthy, one cannot be considered healthy. Similarly, if one is not mentally healthy, one is not able to do anything." (FGD 1, ANC, Pakistan)
	Q10	"Good health means being mentally, socially, emotionally and physically fit." (FGD 2, ANC, India)
Social components of health	Q11	"If there is peace in the home and in the mind, then it is good health." (KII 3, PNC, Pakistan)
	Q12	"If there are fights in the home, this effects a woman's health negatively." (KII 8, PNC, Pakistan)
	Q13	"The surrounding environment and family atmosphere directly affects [physical] health." (FGD 4, PNC, India)
**Ill-health Sub-themes**	**Illustrative quotes**	
Physical symptoms	Q14	"Ill-health means presence of disease." (FGD 4, ANC, India)
	Q15	"Those who are not healthy, their face shows it; they have yellowish complexion and have dark circles around the eyes." (FGD 1, ANC, Pakistan)
	Q16	"Ill-health is like flu, fever and sore throats." (KII 5, PNC, Pakistan)
	Q17	"Poor health means having pain, discomfort, fever and body ache." (KII 4, PNC, Pakistan)
	Q18	"Disease is for example HIV/AIDS, weak bones, weakness." (KII 5, PNC, Pakistan)
Psychological components of ill- health	Q19	"Ill-health is about tension; even if there is a very low level of stress or tension it does great damage to a person." (KII 2, PNC, Pakistan)
	Q20	"Mental health affects physical health. If one is mentally disturbed, won't be able to do anything, even if you are physically fit, you will feel weak." (KII 9, PNC, Pakistan)

For peer review only - http://bmjopen.bmj.com/site/about/guidelines.xhtml

Table 3: Womens' understanding of health and ill-health during and after pregnancy.

Health Sub-theme	Illustrative quotes	
Diet	Q21	"In pregnancy, dietary care is more important to remain healthy, as there is a baby as well." (FGD 4, ANC, India)
	Q22	"One needs to be extra careful and eat more in pregnancy because it is the matter of two lives now." (KII 2, PNC, Pakistan
Medication	Q23	"Good health in pregnancy is when you take your medicine regularly and completely." (KII 4, PNC, Pakistan)
Extra care is required during and after pregnancy for health	Q24	"You have to take really good care of yourself in pregnancy. If you become weak, the complications increase, and the baby will also be weak." (KII 4, PNC, Pakistan)
	Q25	"Pregnant woman must take a nap for one and a half hour to two hours during daytime and try to take more rest at night." (FGD 2, PNC, India)
	Q26	"The most important thing is this that baby is healthy and to have the proper medical care so that both baby and mother stay healthy." (KII 7, PNC, Pakistan)
Psychological components	Q27	"Women should not have any tension during pregnancy." (FDG 3, ANC, India)
Social components	Q28	"If family members are quarrelsome and abusive, it affects both the mother and baby's health." (FGD 2, ANC, India)
**Ill- Health Sub-theme**	**Illustrative quotes**	
Physical symptoms	Q29	"Ill-health is …. abdominal pain, leaking per vagina, vomiting, loss of appetite and dizziness." (FGD 2, ANC, India)
	Q30	"There are many symptoms that are dangerous, like if there is lack of fluids or baby around the baby is not growing as it should be, or if baby is suffering due to negligence." (KII 5, PNC, Pakistan)
	Q31	"Bleeding or swelling is dangerous." (FGD 3, ANC, Pakistan)
Psychological components of health	Q32	"Stress affects health in pregnancy, if there is any stress or tension one cannot eat properly. It also affects the baby's health and our own health too." (KII 4, PNC, Pakistan)
Social components of health	Q33	"If family members are quarrelsome and abusive, it affects both the mother and baby's health." (KII 7, PNC, Pakistan)
	Q34	"The husband comes home after drinking and beats the woman." (FDG 2, ANC, India)
	Q35	"If a husband spends his entire income on drinking, he comes home late and beats his pregnant wife." (FDG 4, ANC, India)
Gender preference of the baby	Q36	"In some families, male gender preference is a major cause of stress and tension for pregnant women." (FDG 1, ANC, India)
	Q37	"This time also I have given birth to a girl child [and] because of this the family atmosphere is not good. My family members are abusing me and because of this I am going through a lot of stress." (FDG 5, PNC, India)

For peer review only - http://bmjopen.bmj.com/site/about/guidelines.xhtml

Table 4: Women's views regarding whether healthcare providers should enquire regarding psycho-social ill-health

Sub-theme		Illustrative quotes
**Psycho-social ill-health is not currently addressed**	Q38	"They do not ask about mental health or the family situation. They just ask about test reports, blood deficiency" (FGD 4, ANC, Pakistan)
	Q39	"They ask in detail about me, about my diet and medicines, they take a complete history and ask about all my previous deliveries, but they never ask about my family environment or mental health." (KII 10, PNC, Pakistan)
	Q40	"They usually don't ask much, and we also do not talk much; they check, give medicine, or sometimes ask, does your husband smoke and is there any disease in the family?" (KII 7, PNC, Pakistan)
**Psycho-social ill-health should be assessed**	Q41	"Yes, they should ask us." (FGD 4, ANC, India)
	Q42	"They should ask such questions, but they do not. They should ask about family conflicts and talk about the solutions." (KII 11, PNC, Pakistan).
	Q43	"If one has some grief, sharing with someone lessens the grief, though it may not solve the problem, but releases stress." (KII 6, PNC, Pakistan)
**Education**	Q44	"Women who are uneducated or unaware might get offended by these questions, others will not mind." (FGD 2, ANC, Pakistan)
	Q45	"People who understand such issues will not react, but others will." (FGD 3, ANC, India)
**Stigma**	Q46	"Some women will not talk about their problems as they are afraid. They think that their husband will know about it if they talk to doctor and will be angry with them." (FGD 4, ANC, Pakistan)
	Q47	"I will feel ashamed to talk about my condition… I can tolerate until it is really necessary." (KII 8, PNC, Pakistan)
	Q48	"Some women don't share. Some women keep everything inside. It also depends upon the woman's personality." (KII 11, PNC, Pakistan)
**Should not be assessed**	Q49	"Doctors should not ask these questions, no, they should not." (KII 5, PNC, Pakistan)
	Q50	"No, doctor has no right to….no doctor has no right to ask such questions." (KII 6, PNC, Pakistan)
	Q51	"A doctor has many patients …Obviously, they don't have time for these details!" (FGD 4, ANC, Pakistan)
**Impact on family**	Q52	"If you ask about the family situation, our community is such that women will be afraid as to why these questions are being asked and they have a fear that in-laws will find out" (FGD 2, ANC, Pakistan)
	Q53	"If the doctor asks in front of our family, that will help change bad behaviours in the family" (FGD 3, ANC, Pakistan)
**Solutions**	Q54	"They should use questions in a way that are focused and clear." (FGD3, ANC, Pakistan)
	Q55	"Do you have any tension? How is your home environment? How is your husband and your family-in-laws behaviour with you?" (KII 10, PNC, Pakistan)

For peer review only - http://bmjopen.bmj.com/site/about/guidelines.xhtml

For peer review only

For peer review only - http://bmjopen.bmj.com/site/about/guidelines.xhtml

LSTM
CENTRE FOR MATERNAL
AND NEWBORN HEALTH

Topic guides for women during and after pregnancy for their views and understanding of maternal health and ill-health

For peer review only

For peer review only - http://bmjopen.bmj.com/site/about/guidelines.xhtml

1. TOPIC GUIDE FOR PREGNANT AND POSTNATAL WOMEN

PROFILE OF WOMEN

Once participants have signed the consent form, ask them to answer these following questions. It will help analyse the qualitative data.

Study setting: ____

Age: ____

Parity: ____

Pregnant status / antenatal or postnatal and number of weeks: _______________________

FORMAT

- Introduce yourself and welcome the group.
- Explain that we are here to learn more about the groups' views on their general views regarding what they understand all types of ill-health to be during and after pregnancy.
- In addition, explain that we are keen to understand what the group in general think regarding questions concerning other types of ill-health that may be a risk factor to pregnancy and how best to approach and ask these questions routinely in their setting.
- Ensure everyone has provided information for the registration log sheet and has a participant information sheet.
- Obtain informed consent for each participant.
- Provide a brief introduction to the relevant ground rules for the key informant interview or focus group discussion:
  (1) The discussion will last a maximum of 1 hour
  (2) The content of the discussion will remain confidential and will not be used outside of the group except for the purposes of the research
  (3) Participants are also requested to keep the discussion confidential after the session
  (4) Participants' names will not be used when reporting the findings
  (5) Participants are encouraged to speak their mind and there are no right or wrong answers
  (6) Participants should not hesitate to disagree with someone else
  (7) But should not talk at once
  (8) The interview will be recorded using a small hand held voice recorder device.
- A voice recorder will be used only to ensure that none of the information from the FGD is lost, and to facilitate analysis of the data from the interviews.

For peer review only

MATERIALS

Topic guide, log, notepad, pens, voice recorders, batteries, information sheet, consent form, transport reimbursement and reimbursement form, refreshments, voice recorders.

FGD QUESTION GUIDE

Reminder to moderator: listen actively and always use probing questions to follow up and obtain further detail from the group (e.g. can you tell me more about that, can you give an example, why is that).

Introduction

We are very interested to hear about your general views regarding what you understand health and ill-health to be for women that you care for during and after pregnancy.

In addition, we are keen to understand how best to ask general questions concerning other types of ill-health that may be a risk factor to the pregnancy. These general questions will include how best to screen for psychological ill-health (depression) or social ill-health (domestic violence or substance misuse) during and after pregnancy.

We would like to understand your perceptions regarding the possible barriers or enabling factors to routinely screen women during and after pregnancy for all types of ill-health (physical, psychological and social) and whether you think women's general health needs are current being met in routine antenatal and postnatal care in your setting.

General questions to ask

1. What is health and what is ill-health during and after pregnancy?

We are interested to know about your general views of what health is and what ill-health in general is for women during and after their pregnancy.

- What do women in your setting consider to be 'good health'?
- What do women in your setting you consider to be 'poor health'?
- In relation to pregnancy: what would good health be and what would poor health be?
- If we consider poor health in pregnancy, what does this mean?
- What kinds of conditions or complaints do pregnant women tend to have?

- Is poor health different in pregnancy and after pregnancy? How?

- Probe: is health only related to physical health?

For peer review only - http://bmjopen.bmj.com/site/about/guidelines.xhtml

- Probe: does ill-health during and after pregnancy also include psychological, and social factors?
- Probe: In your opinion, do women have ill-health during and after their pregnancy? and if so what how does this bother them?

2. How best can a healthcare provider ask questions routinely regarding other forms of ill-health women experience during and after pregnancy?

We are keen to understand how best you think a healthcare provider can ask routinely regarding other forms of ill-health women experience during and after pregnancy, that may be a risk factor to the pregnancy, for example psychological ill-health (depression) or social ill-health (domestic violence or substance misuse)?

Can you tell us your general view and opinion on whether it is acceptable for healthcare providers to make enquiries regarding not just physical but also psychological and social ill-health during routine antenatal and postnatal care?

- Probe: What do you think about women being asked about their perception of their health in pregnancy?
- What kinds of questions do you think pregnant women should be asked to assess how they feel about their health?

- What do you think women would think regarding being asked about their mental well-being or social factors?
- Is it acceptable to be asked about this? Why? Why not?
- How do you think women should be asked these questions?

3. We would like to understand your general perceptions regarding the possible barriers or enabling factors to routinely screen women during and after pregnancy for all types of ill-health (physical, psychological and social).

- In your community, how is pregnancy perceived?
- Are pregnant women treated any differently? In what way?
- When women are pregnant do they expect to feel ill?
- What kinds of conditions or complaints are perceived as 'routine' during pregnancy?
- What conditions or complaints are not considered a 'routine' part of pregnancy?
- How do women deal with minor common discomforts s during pregnancy?

For peer review only - http://bmjopen.bmj.com/site/about/guidelines.xhtml

- How do women deal with those that are not considered a common part of pregnancy?

4. Do you think women's general health needs are current being met in routine antenatal and postnatal care in your setting?

We would like to understand more about whether the existing health care system in your general opinion, meets the health needs of women during and after pregnancy in your community?

Probe: To what extent do you think the health facility meets womens' needs during and after pregnancy? In your opinion does the health system in general provide good quality to women during and after pregnancy?

Probe: What do you think should be done to improve provision of services for women during and after pregnancy?

In summary:
Ask if participants would like to add further comments.

Bring the meeting to a close by summarizing the main points.

Do not forget to say thank you to the participants for their time and active participation.

For peer review only - http://bmjopen.bmj.com/site/about/guidelines.xhtml

COREQ (COnsolidated criteria for REporting Qualitative research) Checklist

A checklist of items that should be included in reports of qualitative research. You must report the page number in your manuscript where you consider each of the items listed in this checklist. If you have not included this information, either revise your manuscript accordingly before submitting or note N/A.

Topic	Item No.	Guide Questions/Description	Reported on Page No.
**Domain 1: Research team and reflexivity**			
Personal characteristics			
Interviewer/facilitator	1	Which author/s conducted the interview or focus group?	
Credentials	2	What were the researcher's credentials? E.g. PhD, MD	
Occupation	3	What was their occupation at the time of the study?	
Gender	4	Was the researcher male or female?	
Experience and training	5	What experience or training did the researcher have?	
Relationship with participants			
Relationship established	6	Was a relationship established prior to study commencement?	
Participant knowledge of the interviewer	7	What did the participants know about the researcher? e.g. personal goals, reasons for doing the research	
Interviewer characteristics	8	What characteristics were reported about the inter viewer/facilitator? e.g. Bias, assumptions, reasons and interests in the research topic	
**Domain 2: Study design**			
Theoretical framework			
Methodological orientation and Theory	9	What methodological orientation was stated to underpin the study? e.g. grounded theory, discourse analysis, ethnography, phenomenology, content analysis	
Participant selection			
Sampling	10	How were participants selected? e.g. purposive, convenience, consecutive, snowball	
Method of approach	11	How were participants approached? e.g. face-to-face, telephone, mail, email	
Sample size	12	How many participants were in the study?	
Non-participation	13	How many people refused to participate or dropped out? Reasons?	
Setting			
Setting of data collection	14	Where was the data collected? e.g. home, clinic, workplace	
Presence of non-participants	15	Was anyone else present besides the participants and researchers?	
Description of sample	16	What are the important characteristics of the sample? e.g. demographic data, date	
Data collection			
Interview guide	17	Were questions, prompts, guides provided by the authors? Was it pilot tested?	
Repeat interviews	18	Were repeat inter views carried out? If yes, how many?	
Audio/visual recording	19	Did the research use audio or visual recording to collect the data?	
Field notes	20	Were field notes made during and/or after the inter view or focus group?	
Duration	21	What was the duration of the inter views or focus group?	
Data saturation	22	Was data saturation discussed?	
Transcripts returned	23	Were transcripts returned to participants for comment and/or	

For peer review only

Topic	Item No.	Guide Questions/Description	Reported on Page No.
		correction?	
**Domain 3: analysis and findings**			
Data analysis			
Number of data coders	24	How many data coders coded the data?	
Description of the coding tree	25	Did authors provide a description of the coding tree?	
Derivation of themes	26	Were themes identified in advance or derived from the data?	
Software	27	What software, if applicable, was used to manage the data?	
Participant checking	28	Did participants provide feedback on the findings?	
Reporting			
Quotations presented	29	Were participant quotations presented to illustrate the themes/findings? Was each quotation identified? e.g. participant number	
Data and findings consistent	30	Was there consistency between the data presented and the findings?	
Clarity of major themes	31	Were major themes clearly presented in the findings?	
Clarity of minor themes	32	Is there a description of diverse cases or discussion of minor themes?	

Developed from: Tong A, Sainsbury P, Craig J. Consolidated criteria for reporting qualitative research (COREQ): a 32-item checklist for interviews and focus groups. *International Journal for Quality in Health Care*. 2007. Volume 19, Number 6: pp. 349 – 357

Once you have completed this checklist, please save a copy and upload it as part of your submission. DO NOT include this checklist as part of the main manuscript document. It must be uploaded as a separate file.

For peer review only

BMJ Open

“Good health means being mentally, socially, emotionally and physically fit”: Women’s understanding of health and ill-health during and after pregnancy in India and Pakistan: a qualitative study

Journal:	*BMJ Open*
Manuscript ID	bmjopen-2018-028760.R2
Article Type:	Research
Date Submitted by the Author:	08-Oct-2019
Complete List of Authors:	McCauley, Mary; Liverpool School of Tropical Medicine, Centre for Maternal and Newborn Health<br>Avais, Ayesha ; Child Advocacy International Pakistan<br>Agrawal, Ritu; Vardhman Mahavir Medical College and Safdarjung Hospital<br>Saleem, Shumaila; Child Advocacy International Pakistan<br>Zafar, S; Fazaia Medical College<br>van den Broek, Nynke; Liverpool School of Tropical Medicine, Centre for Maternal and Newborn Health
**Primary Subject Heading**:	Obstetrics and gynaecology
Secondary Subject Heading:	Evidence based practice, Global health
Keywords:	Maternal medicine < OBSTETRICS, Antenatal care, Postnatal care, Maternal morbidity, Understanding of health and ill-health

SCHOLARONE™ Manuscripts

"Good health means being mentally, socially, emotionally and physically fit": Women's understanding of health and ill-health during and after pregnancy in India and Pakistan: a qualitative study

Authors:

MCCAULEY, Mary[1]*

AVAIS, Ayesha Rasheeda[2]

AGRAWAL, Ritu [3]

SALEEM, Shumaila[2]

ZAFAR, Shamsa[4]

VAN DEN BROEK, Nynke[1]

[1]Centre for Maternal and Newborn Health, Liverpool School of Tropical Medicine, United Kingdom

[2]Child Advocacy International Pakistan, Islamabad, Pakistan

[3]Safdarjung Hospital, New Delhi, India

[4]Fazaia-Medical College, Air University, Islamabad, Pakistan

*Corresponding author: mary.mccauley@lstmed.ac.uk

Word count: 3669

Ethical Approval: The Liverpool School of Tropical Medicine, Liverpool, United Kingdom, Safdarjung Hospital, New Delhi and the National Bioethics Committee, Islamabad, Pakistan all granted ethical approval.

Disclosure of Funding Received: This study was funded by a Research Development Fund (RDF160301MMC11) from the Liverpool School of Tropical Medicine, Liverpool, United Kingdom.

Short Title:

Women's views on health and ill-health during and after pregnancy in India and Pakistan.

Keywords: Health, ill-health, antenatal care, postnatal care, pregnancy, maternal morbidity.

For peer review only

Abstract

Objective

To explore what women, consider health and ill-health to be, in general, and during and after pregnancy. Women's views on how to approach and discuss all components of ill-health as part of routine screening during and after pregnancy, including mental health and social morbidities were also explored.

Setting

A public hospital in New Delhi, India and a public hospital in Islamabad, Pakistan.

Participants

130 women attending for routine antenatal or postnatal care at the study healthcare facilities.

Interventions

Data collection was conducted using focus group discussions and key informant interviews. Transcribed interviews coded by topic and grouped into categories. Thematic framework analysis was undertaken to identify emerging themes.

Results

Most women are aware that health is a multidimensional concept, not simply physical symptoms or identified medical disease. Concepts of "good health" included a nutritious diet; having an ideal weight; absence of disease; and a supportive family environment. Ill-health consisted of physical symptoms and disease; stress and tension; and domestic violence and alcohol use in the family. Women are aware that the health or ill-health of the mother and baby are interlinked. In addition to a full physical assessment, most women welcome mental and social ill-health enquiries. Reported barriers to routine enquiry regarding mental and social ill-health included women's perceptions that these issues are 'personal'; that healthcare providers do not have the time and/or cannot provide further care, even if mental or social ill-health is disclosed.

Conclusions

Women have a good understanding of the comprehensive nature of health and ill-health during and after pregnancy. Women report that enquiry regarding mental and social ill-health is currently not part of routine maternity care, but most welcome such an assessment. There is a need to understand how to support healthcare providers to screen for all aspects of

For peer review only - http://bmjopen.bmj.com/site/about/guidelines.xhtml

maternal morbidity (physical, mental and social); and for healthcare providers to be enabled to provide evidence based care for women if ill-health is disclosed.

Keywords

Health, ill-health, maternal health, women, antenatal care, postnatal care, qualitative research, perceptions.

Word count: 318

Strengths and limitations of this study

- We conducted a qualitative study to obtain women's own opinions on what they consider health and ill-health to be during and after pregnancy.
- We also assessed women's views on routine screening for mental and social ill-health as components of comprehensive maternity care.
- We interviewed 130 women attending for routine antenatal or postnatal care in secondary level public hospitals in India and Pakistan.
- We only interviewed women in urban settings and excluded women living in rural communities who may have alternative perspectives or different insights.

For peer review only

Introduction

All women have the right to the highest attainable standard of health and well-being including physical, mental and social aspects [1,2]. The Sustainable Development Goals (SDG) developed by the United Nations and endorsed by 193 countries worldwide, envision that that every woman in every setting has an equal chance to survive and thrive [3]. SDG 3 is to "ensure healthy lives and promote well-being for all' [3]. The Global Strategy strives for a world in which every mother can enjoy a wanted and healthy pregnancy and childbirth and that pregnancy is an opportunity for a woman to receive care to "thrive" [1]. Currently this is not the case in many low-and middle- income countries (LMIC). As part of the need to develop innovative strategies to achieve SDG 3, there has been a renewed focus on maternal ill-health or "maternal morbidity" in terms of definition, measurement, and interventions [4-10]. The burden of disease of the various aspects of health (physical, mental and social) during and after pregnancy and their impact on women and/or pregnancy outcomes are currently not well documented in a comprehensive, standardised approach [4,5]. It is suggested that although maternal ill-health occurs across all countries, cultures, religions, socioeconomic status, and ages, the burden of maternal morbidity is expected to be higher in LMIC with an estimated three out of four women suffering at least one form of maternal morbidity during or after their pregnancy [4,5].

Maternal ill-health or morbidity is a complex and broad concept and can be understood and described in various ways depending on different perspectives [8-10]. For example, women, men, families, communities, healthcare providers and policy makers may have mixed views and perceptions on what maternal ill-health is and the effect or risk of maternal morbidity during and after pregnancy for the women and/or her baby [9]. Maternal morbidity has been defined as "any health condition that is attributed to or aggravated by pregnancy and childbirth which has a negative impact on the woman's wellbeing" [8]. However, there is still a lack of understanding and lack of data regarding what women themselves consider ill-health to be (physical and/or mental and/or social) and their experiences (subjective measures) compared to clinical findings (objective measures) when assessed and defined as "morbidity" by a healthcare provider; and there is especially a lack of data from women living in LMIC [4,5, 9]. The WHO have recently highlighted the need for qualitative studies that explore women's lived experiences of ill-health and perceptions of their health and health needs during and

For peer review only - http://bmjopen.bmj.com/site/about/guidelines.xhtml

after pregnancy [8-10]. This study therefore sought to explore what women attending for routine antenatal care (ANC) or postnatal care (PNC) at healthcare facilities in India and Pakistan consider health and ill-health to be in general, and, what they consider health and ill-health to be, during and after pregnancy. As a component of maternal ill-health, women's views on how best to approach aspects of mental ill-health (such as depression and/or anxiety) and social ill-health (such as exposure to violence and/or substance misuse), were assessed.

Materials

Study design

Experienced female researchers (AA, RA, SS) interviewed a total of 130 women. Data collection was conducted using focus group discussions (FGD) to interview 60 women attending for routine ANC or PNC in the outpatient clinics at a large public hospital in New Delhi, India. FDGs and key informant interviews were used to interview 70 women attending for routine ANC or PNC in the outpatient clinics at a large public hospital in Islamabad, Pakistan. The sampling frame included (1) women who were pregnant or who have given birth within the past six weeks and (2) women who above the legal age of consent. Purposive sampling was used to approach women attending each study setting healthcare facility for routine ANC or PNC care. Women were only included once in the study. All interviews and FGDs were held in quiet locations away from the clinical areas at the relevant hospitals.

Participants and settings

All women attending for routine ANC or PNC in the study healthcare facilities were invited to participate. The study settings were two large public urban hospitals serving women with low-lower middle socio-economic status; and with varied education levels [4]. Similar proportions of women were included from four different stages of pregnancy and FGDs included women within the same assessment stage of pregnancy (first half of pregnancy (≤20 weeks); second half of pregnancy (>20 weeks); early postnatal stage (day 1-7); or late postnatal stage (week 2-6). Women were purposively selected and recruited sequentially until the number of anticipated FGD and key informant interviews was reached, and data saturation was achieved.

For peer review only - http://bmjopen.bmj.com/site/about/guidelines.xhtml

Topic guide

A topic guide was developed to guide the interviews and was piloted in the United Kingdom with clinical researchers from LMIC (Supplementary document 1). The topic guide was then translated into the local study setting language, back-translation was conducted, and the topic guide was further piloted in each study setting. As a result, the topic guide was refined; for example, some wording was changed in the local language and the introduction was amended to ensure that the women were aware that we sought to assess their general views and not their own personal experiences (if any) of different types of ill-health. The topic guide served as a flexible tool to facilitate the facilitators in obtaining the women's opinions whilst ensuring that the interview remained on topic. The topic guide also acted as a cue to ask more probing questions to further understand women' beliefs and perceptions. The topic guide included three main subject areas:

1) perception and understanding of health and ill-health in general;

2) perception and understanding of health and ill-health during and after pregnancy;

3) views on enquiry regarding mental and social ill-health during and after pregnancy; and suggestions on how best to approach all components of health.

Data Collection

Prior to interview, all eligible women were approached and given verbal and written information regarding the study including a brief overview of the research aims and interview questions. Appointments for FGD or key informant interviews were then scheduled for a later time convenient for the women. The FGD and key informant interviews were conducted in the local language (India-Hindi and Pakistan-Urdu) by the local female trained and experienced researchers (AA, RA, SS). On average the FGDs lasted 60-90 minutes, and the key informant interviews lasted 30 minutes. All FGD and key informant interviews were conducted face-to-face, recorded on a digital recording device and transcribed in the local language upon completion by the local researchers (AA, RA, SS). All efforts were made to emphasise confidentiality for interviews to ensure women were confident in giving honest answers and member checking was used to assure correct interpretation. Interviewing women from different stages of pregnancy, different backgrounds, and different countries broadened the scope of the topic and enabled transferability to be achieved to an extent.

For peer review only - http://bmjopen.bmj.com/site/about/guidelines.xhtml

For peer review only

Data analysis

In each study setting, transcribed interviews were translated into English and initially manually open coded following data familiarisation (reading and re-reading of the transcripts several times) by the local researchers (RA in India; AA and SS in Pakistan). In a sub-sample of cases, and prior to translation, participants were asked to review the coded transcripts to verify the accuracy and appropriateness of the applied code. Using thematic analysis, codes were identified from the transcripts and grouped into categories independently by the local researchers, enabling the first abstraction of data in each setting [11]. Thematic framework analysis of the categories was then undertaken. The separate results were then brought together and refined to agree on the key themes. All of the coded data was reviewed by other members of the research team (MMc in India and SZ in Pakistan) to ensure consistency and to check for interrater reliability. In addition, all members of the research team reviewed, and critiqued emerging themes and sub-themes and any discrepancies were discussed, and consensus agreed. The reporting of the results adheres to the COREQ guidelines [12].

Patient and Public Involvement

No patient involved.

Ethics

The Liverpool School of Tropical Medicine, Liverpool, United Kingdom, granted full ethical approval (LSTM 16-054RS). Ethical approval was also obtained from the Research and Ethics Committee, Vardhman Mahavir Medical College and Safdarjung Hospital, New Delhi, India (ICE/SJH/VMMC/Project/October-2016/688); and the National Bioethics Committee, Islamabad, Pakistan (No.4-87/16/NBC-159-Addendum/RDC/3634). Written informed consent was obtained from each woman who participated in the study.

Results

A total of 130 women were interviewed (**Table 1**). Three women invited to participate declined due to time constraints. A total of 83 women were pregnant at the time of interview and 47 had given birth with the past six weeks. Most women were either in their first pregnancy or had one previous pregnancy. A minority of women had more than two previous

For peer review only - http://bmjopen.bmj.com/site/about/guidelines.xhtml

pregnancies. The age range was from 20-36 with a mean of 26 years old. There were no distinct differences in emerging themes or sub-themes from women from India or Pakistan. Therefore, all quotes illustrating the results are combined for both countries and displayed in tables and linked to the narrative text.

What is health?

Women perceived "good health" in general as having a good diet; having an ideal weight; absence of physical disease; absence of stress and tension; and a supportive family environment (Table 2). Most women expressed that the ability to have a good diet would result in good health (Table 2, Q1-Q3). Weight was mentioned in India and Pakistan, however, some women's perception of this differed (Table 2, Q4-Q5). In both settings, women reported that health meant lack of disease and good energy levels (Table 2, Q6-Q7). Most women reported that health was not just physical health but includes mental health (Table 2, Q8-Q10). Most women highlighted that a supportive environment with family were important for good health (Table 2, Q11-Q13).

What is ill-health?

The emerging themes related to perceived ill-health included; physical symptoms and disease; tension; and an abusive and stressful home environment. Many women expressed that ill-health was "pain, flu, fever, sore throat, weakness, body ache, headache, and swelling" (Table 2, Q14-Q17). Ill-health was also described in terms of disease, for example HIV/AIDS (Table 2, Q18). Mental ill-health was often considered a health concern and stress and tension was reported by many women as a component of or factor leading to mental ill-health (Table 2, Q19-Q20).

What is health during and after pregnancy?

During and after pregnancy a nutritious diet, medication, rest, extra care, and a peaceful home environment were mentioned as important. Most women reported that diet is even more important when a woman is pregnant (Table 3, Q21-Q22). Many women mentioned that pregnant women needed medication and more care from the family and healthcare providers (Table 3, Q23-Q25). Many women understood that their health was "lack of tension" and that their (or ill-health) was linked to that of their baby (Table 3, Q26-Q28).

For peer review only - http://bmjopen.bmj.com/site/about/guidelines.xhtml

What is ill-health during and after pregnancy?

Perceived ill-health during and after pregnancy included the perception that pregnancy is a "disease", and that there was "no health in pregnancy", as all "the energy goes to the baby and the mother becomes weaker". Women also considered some physical symptoms and discomfort as "normal" during pregnancy. This included nausea and vomiting, back pain and difficulty sleeping. However, many women reported that ill-health during and after pregnancy was "vomiting, high blood pressure, bleeding, swelling, abdominal pain" (Table 3, Q29). Many women were aware that these symptoms could be dangerous (Table 3, Q30-Q31). Stress and tension were considered important components of ill-health by most women (Table 3, Q32). An abusive family environment was highlighted as examples of social ill-health in women during and after pregnancy (Table 3, Q33-Q35). There was some discussion regarding gender preference, and it was suggested by a minority of women that the gender of a baby created stress and tension for women (Table 3, Q36-Q37).

Should healthcare providers ask about mental and social ill-health?

An emerging theme regarding what women consider health and ill-health to be, was the importance of mental and social health and well-being. Women described mental ill-health such as "depression", "weeping", stress", "tension", "grief", "loss of appetite" and "unhappiness". Women reported social concerns such as "fights in the home", "domestic violence", "poor family environment", "quarrels and verbal abuse", "family or husband alcohol misuse", and an "expectation of a male child". Women were therefore asked questions regarding how best to approach and address the concepts of mental and social ill-health for women during and after pregnancy. Most women reported that healthcare providers ask pregnant women questions regarding physical ill-health only (Table 4, Q38-Q40). Most women felt that there was a lack of enquiry from healthcare providers regarding mental and social ill-health during and after pregnancy. Most women felt that healthcare providers could and should ask women about their mental and social health (Table 4, Q41-Q43). Many women said that it would be beneficial to speak to their healthcare provider if they were feeling "stressed" and "under tension" as the healthcare provider might be able to provide general support and advice. However, a few women explained these types of questions might be considered unacceptable, because of lack of education and understanding

For peer review only - http://bmjopen.bmj.com/site/about/guidelines.xhtml

among some women (Table 4, Q44-Q45). There was some concern that some women would prefer not to talk about their mental and social ill-health as they may feel afraid, ashamed or uncomfortable (Table 4, Q46-Q48). A small minority of women in Pakistan felt it was not the role of the healthcare provider to ask questions regarding mental and/or social health, as this was their “personal life”. Women discussed the repercussions from husbands and family if domestic violence during and after pregnancy would be disclosed by a woman to a healthcare provider (Table 4, Q49-Q51). There were concerns that a woman would not be allowed to access care again. There was a lot of discussion about this with sometimes conflicting opinions; ranging from it not being right for a woman to disclose domestic violence, or mental ill-health such as stress and tension; to a consideration that healthcare providers should ask and would provide help. A few women said that if they themselves were asked questions regarding depression or domestic violence, they would seek care from another healthcare provider to avoid these questions. Many women recognised that there were existing barriers for healthcare providers which made it difficult for them to ask women questions regarding mental and social ill-health during and after pregnancy (Table 4, Q52-Q53). Such barriers included the perceived heavy workload of healthcare providers and lack of time to explore mental and/or social concerns (Table 4, Q51). Other women explained that “they can ask, but what can they do?” and that “nothing can be done” to help. Some women did offer suggestions of how healthcare providers could best ask questions regarding mental and social ill-health during and after pregnancy. A more empathic approach to women were suggested including politeness and friendliness were suggested. Confidentiality was highlighted as important by many women. Some women suggested that healthcare providers need not go into such detailed questions but keep any questions simple (Table 4, Q54-Q55).

Discussion

Statement of principal findings

Most women who took part in this study had clear opinions and had a good understanding that health includes more than physical components of health and that the health of the mother was linked to that of the baby. Good diet, healthy weight, rest and a supportive home environment contributed to good health; whereas physical disease symptoms, complications of pregnancy, as well as stress, tension and an abusive family environment contributed to ill-

For peer review only - http://bmjopen.bmj.com/site/about/guidelines.xhtml

health. Women were aware of physical common discomforts and of more severe complications and dangers signs, as ill-health related to pregnancy. Women considered mental and social ill-health as important but reported that healthcare providers do not currently ask women how they are feeling or ask after their mental nor social health and wellbeing . However, most women would be happy if healthcare providers did so. A minority of women considered mental and social health concerns as “personal” and reported that there may be barriers to asking such questions.

Strengths of the study

To the best of our knowledge, this is the first study from two LMIC settings (India and Pakistan) to obtain women’s own opinions on what they might consider health and ill-health to be in general and during and after pregnancy. A range of women from four different stages of pregnancy were interviewed resulting in a wide spectrum of responses. Most women approached welcomed the discussion surrounding health, and ill-health during and after pregnancy, and were keen to contribute to solutions in their settings regarding healthcare providers roles in routine enquiry regarding other components of health including mental and social aspects, and not just physical health.

Limitations of the study

This study population included women attending for routine ANC or PNC in public secondary level healthcare facilities in urban settings and excludes other women who may have alternative perspectives or different insights. There is a need to assess the views of women in other LMIC and including community and rural settings, as these women may have different perceptions and understandings.

How does this study relate to other literature?

There is lack of literature regarding the understanding of what women consider health and ill-health to be during and after pregnancy in India and Pakistan. There is also debate that the current definition of health [2] is out-dated and needs to be re-formulated to consider health in a context of functionality, capacity, adaptability and the ability to perform activities of daily living despite having a disease or disability [13]. There have been various proposals to develop

For peer review only - http://bmjopen.bmj.com/site/about/guidelines.xhtml

the definition of health using the concept of “health, as the ability to adapt and to self-manage” but with a continued emphasis on the importance of the three domains of health: physical, mental, and social [13]. Aspects of physical ill-health in women during and after pregnancy in LMIC settings are well documented and there are many recommendations and interventions developed to diagnose and manage these conditions; for example, anaemia, haemorrhage, hypertensive disorders of pregnancy, infection, and complications of obstructed labour [14-18]. With regards to mental and social ill-health in women during and after pregnancy in LMIC, there is data to demonstrate that depression and domestic violence represent a large burden of ill-health and have a negative impact on a woman’s health and well-being [19-22]. However, to date, clinical practice in low resource settings rarely takes mental or social ill-health into account when assessing a woman’s health needs during and after pregnancy [23-24]. However, this study demonstrates that most women would welcome such an assessment but report that their healthcare providers do not current ask; and even if they did, some women report that “nothing that can be done”. This may be due to a shortage of human resources but also because of healthcare providers’ perceptions that these issues are not “ill-health” per se and because of perceived cultural sensitivities related to mental ill-health and -social concerns[4,23-26].

It is well recognised that maternal physical, mental and social ill-health is linked to newborn babies’ health and women in our study demonstrated an understanding that all aspects of ill-health are associated with poor fetal and newborn health outcomes [27-30]. The detrimental impact of mental and social morbidity on the overall health and well-being of mothers (and their babies) during and after pregnancy has resulted in public health policy in high income countries such as the United Kingdom (UK), where enquiry regarding of mental ill-health (depression, anxiety) and social circumstances (exposure to domestic violence and/or substance misuse), is routinely conducted during and after pregnancy [31]. The WHO have produced clinical and policy guidelines on how to comprehensively care for women during and after pregnancy including identification, safety assessment and planning, communication and clinical skills, documentation and provision of referral pathways for mental and social ill-health [32]. However, the feasibility of implementation and acceptability of this guidance in countries, such as India and Pakistan are currently uncertain. There is debate as to the cadre of healthcare providers most suitable to undertake routine enquiry regarding for and/or

For peer review only - http://bmjopen.bmj.com/site/about/guidelines.xhtml

manage mental and social ill-health in LMIC [23,24,33]. In many high-income countries, specially trained midwives routinely assess, screen, support and provide further referral for women with mental and social morbidity [31]. This study shows that most women are keen to be asked questions regarding their mental and social well-being during and after pregnancy, as part of routine maternity care. However, a minority of women do not wish to be asked.

Currently, there is good coverage and uptake of antenatal care, but content needs to be adapted to ensure good quality comprehensive care that is respectful, integrated and delivers physical, mental and social care, with agreed country specific additional content. Although, mental and social ill-health is clearly a real matter of concern for women, this study was not designed to explore this in further detail. Further research is needed to explain in more depth what these problems are, and who and how they can be addressed. It is important that healthcare providers are supported to provide good quality care for women and that this goes beyond simply the physical aspects of health and is inclusive of mental and social well-being.

Conclusion

Women reported that health and ill-health is not just physical but also includes mental and social components. Maternal ill-health is common among women attending for antenatal and postnatal care [4]. Women are increasingly accessing care during and after pregnancy and there is a window of opportunity to adapt and amend available care packages to include comprehensive enquiry regarding and, where needed, support for mental and social ill-health [4, 10]. This study demonstrates that most women are keen to be asked questions regarding their mental and social well-being and provides simple suggestions that healthcare providers can use to improve the enquiry regarding mental and social ill-health among women during and after pregnancy. There is a need for further research to help develop interventions and deliver maternity care in a way that meets the health needs of women in a comprehensive and holistic manner, including mental and social components [4, 5].

For peer review only - http://bmjopen.bmj.com/site/about/guidelines.xhtml

Abbreviations

ANC: antenatal, **FGD**: Focus group discussion, **LMIC**: Low and middle-income country, **LSTM**: Liverpool School of Tropical Medicine, **SDG**: Sustainable Development Goals, **PNC:** postnatal, **UK**: United Kingdom, **WHO**: World Health Organization

Declarations

Ethics approval and consent to participate

The Liverpool School of Tropical Medicine, Liverpool, United Kingdom, granted full ethical approval (LSTM 16-054RS). Ethical approval was also obtained from the Research and Ethics Committee, Vardhman Mahavir Medical College and Safdarjung Hospital, New Delhi, India (ICE/SJH/VMMC/Project/October-2016/688); and the National Bioethics Committee, Islamabad, Pakistan (No.4-87/16/NBC-159-Addendum/RDC/3634). Written informed consent was obtained from each woman who participated in the study.

Consent for publication

Written informed consent was obtained from all women in the study, including audio-recording and the use of excerpts in publications and reports.

Availability of data and material

The datasets used and analysed during the current study are available from the corresponding author on reasonable request.

Competing interests

The authors declare that they have no competing interests.

Funding

Disclosure of funding received: This study was funded by the Liverpool School of Tropical Medicine Research Development Fund (RDF160301MMC11).

Author's contributions

MMC conceived the study idea, design and developed the topic guide. SZ co-ordinated and supervised the research activities in Pakistan. AA, SS and RA conducted the interviews, transcription, translations and data analysis. MMc co-ordinated and supervised the overall research activities, interpreted and presented the results and wrote the manuscript. NvdB

For peer review only - http://bmjopen.bmj.com/site/about/guidelines.xhtml

has reviewed the data, interpreted the results and contributed to the manuscript. All authors have read, edited and approved the final manuscript for submission.

Acknowledgements

The authors would like to thank Professor Pratima Mittal, Professor Jyotsna Suri and Dr Mansi for their support with ethics applications and data collection in India. A huge thank you to all the women who participated in this study.

For peer review only

For peer review only - http://bmjopen.bmj.com/site/about/guidelines.xhtml

References

1. United Nations. Global Strategy for Women's, Children's and Adolescents' Health, 2016-2030. New York: United Nations; 2015. http://www.who.int/life-course/partners/global-strategy/en/
2. World Health Organization. Preamble to the Constitution of the World Health Organization as adopted by the International Health Conference, New York, 19-22 June 1946. http://www.who.int/about/definition/en/print.html
3. United Nations. Sustainable Development Goals. http://www.un.org/sustainabledevelopment/sustainable-development-goals/
4. McCauley M, Madaj B, White SA, Dickinson F, Bar-Zev S, Aminu M, Godia P, Mittal P, Zafar S, van den Broek N. Burden of physical, psychological and social ill-health during and after pregnancy among women in India, Pakistan, Kenya and Malawi. BMJ Glob Health. 2018 May 3;3(3):e000625. doi: 10.1136/bmjgh-2017-000625. PubMed PMID: 29736274; PubMed Central PMCID: PMC5935159.
5. Zafar S, Jean-Baptiste R, Rahman A, Neilson JP, van den Broek N. Non-Life Threatening Maternal Morbidity: Cross Sectional Surveys from Malawi and Pakistan. PLoS ONE. 2015;10(9):e0138026.
6. Say L, Souza JP, Pattinson RC, for the WHO working group on Maternal Mortality and Morbidity classifications. Maternal near miss - towards a standard tool for monitoring quality of maternal health care. Best Pract Res Clin Obstet Gynaecol. 2009;23(3):287-96.
7. Chou D, Tunçalp Ö, Firoz T, Barreix M, Filippi V, von Dadelszen P, van den Broek N, Cecatti JG, Say L. Constructing maternal morbidity – towards a standard tool to measure and monitor maternal health beyond mortality. BMC Pregnancy Childbirth. 2016;16:45.
8. Barreix M , Barbour K, McCaw-Binns A, Chou D, Petzold M, Gichuhi GN, Gadama L, Taulo F, Tunçalp Ö, Say L (2018). Standardizing the measurement of maternal morbidity: Pilot study results. Int J Gynecol Obstet, 141: 10-19.
9. Say L, Chou D. (2018), Maternal morbidity: Time for reflection, recognition, and action. Int J Gynecol Obstet, 141: 1-3.
10. Filippi V, Chou D, Barreix M, Say L. (2018), A new conceptual framework for maternal morbidity. Int J Gynecol Obstet, 141: 4-9.

For peer review only - http://bmjopen.bmj.com/site/about/guidelines.xhtml

11. Firoz T, Chou D, von Dadelszen P, Agrawal P, Vanderkruik R, Tunçalp O, Magee LA, van den Broek N, Say L. Measuring maternal health: focus on maternal morbidity. Bull World Health Organ. 2013;91(10):794-6.
12. Braun V, Clarke V. Using thematic analysis in psychology. Qual Res Psychol. 2006;3(2):77-101.
13. Equator Network. Consolidated criteria for reporting qualitative research (COREQ): a 32-item checklist for interviews and focus groups. http://www.equator-network.org/reporting-guidelines/coreq/
14. Huber M, Knottnerus JA, Green L, van der Horst H, Jadad AR, Kromhout D, Leonard B, Lorig K, Loureiro MI, van der Meer JW, et al. How should we define health? BMJ. 2011;343:d416
15. World Health Organization. The WHO Application of ICD-10 to deaths during pregnancy, childbirth and the puerperium: ICD-MM. Geneva: World Health Organization; 2012. http://www.who.int/reproductivehealth/publications/monitoring/9789241548458/en/
16. Abalos E, Cuesta C, Grosso AL, Chou D, Say L. Global and regional estimates of pre-eclampsia and eclampsia: a systematic review. Eur J Obstet Gynecol Reprod Biol. 2013;170(1):1-7
17. Calvert C, Thomas SL, Ronsmans C, Wagner KS, Adler AJ, Filippi V. Identifying regional variation in the prevalence of postpartum haemorrhage: a systematic review and meta-analysis. PLoS ONE. 2012;7(7):e41114.
18. World Health Organization. Haemoglobin concentrations for the diagnosis of anaemia and assessment of severity. Geneva: World Health Organization; 2011. http://www.who.int/vmnis/indicators/haemoglobin.pdf
19. Velu PP, Gravett CA, Roberts TK, Wagner TA, Zhang JSF, Rubens CE, Gravett MG, Campbell H, Rudan I. Epidemiology and aetiology of maternal bacterial and viral infections in low- and middle-income countries. J Glob Health. 2011;1(2):171-88.
20. World Health Organization. Maternal Mental Health; 2017. http://www.who.int/mental_health/maternal-child/maternal_mental_health/en/
21. Vos T, Barber RM, Bell B, Bertozzi-Villa A, Biryukov S, Bollger I and Global Burden of Disease Study 2013 Collaborators.. Global, regional, and national incidence, prevalence, and years lived with disability for 301 acute and chronic diseases and injuries in 188

For peer review only - http://bmjopen.bmj.com/site/about/guidelines.xhtml

countries, 1990-2013: a systematic analysis for the Global Burden of Disease Study 2013. Lancet. 2015;386(9995):743-800.

22. Shrivastava SR, Shrivastava PS, Ramasamy J. Antenatal and postnatal depression: a public health perspective. J Neuros Rural Prac. 2015;6(1):116-9.

23. McCauley M, Brown A, Ofosu B, van den Broek N. "I just wish it becomes part of routine care?":Healthcare providers' knowledge, attitudes and perceptions of screening for maternal mental health during and after pregnancy. BMC Psychiatry 2019; (19): 279

24. McCauley M, Head J, Lambert J, Zafar S, van den Broek, N. "Keeping family matters behind closed doors": Healthcare providers' perceptions and experiences of identifying and managing domestic violence during and after pregnancy. BMC Pregnancy Childbirth. 2017;(17)1:318.

25. World Health Organization. Understanding and addressing violence against women. Geneva: World Health Organization; 2012. http://apps.who.int/iris/bitstream/10665/77432/1/WHO_RHR_12.36_eng.pdf

26. World Health Organization. Global and regional estimates of violence against women: prevalence and health effects of intimate partner violence and non-partner sexual violence Geneva: World Health Organization; 2013. http://apps.who.int/iris/bitstream/10665/85239/1/9789241564625_eng.pdf?ua=1

27. World Health Organization. Framing the health workforce agenda for the Sustainable Development Goals: Biennium report 2016-2017 WHO Health Workforce. Geneva: World Health Organization; 2017 http://www.who.int/hrh/BienniumReportRevised2017.pdf

28. Gelaye B, Rondon MB, Araya R, Williams MA. Epidemiology of maternal depression, risk factors, and child outcomes in low-income and middle-income countries. Lancet Psychiatry. 2016;3(10):973.

29. Zakar R, Nasrullah M, Zakar MZ, Ali H. The association of intimate partner violence with unintended pregnancy and pregnancy loss in Pakistan. Int J Gynecol Obstet. 2016;133:26-31.

30. Satyanarayana VA, Lukose A, Srinivasan K. Maternal mental health in pregnancy and child behavior. Indian J Psychiatry. 2011;53(4):351.

For peer review only

For peer review only - http://bmjopen.bmj.com/site/about/guidelines.xhtml

31. National Institute for Health and Care Excellence. Antenatal and postnatal mental health: clinical management and service guidance. London: NICE; 2015. https://www.nice.org.uk/guidance/cg192

32. World Health Organization. WHO recommendations on antenatal care for a positive pregnancy experience. Geneva: World Health Organization; 2016. http://www.who.int/reproductivehealth/publications/maternal_perinatal_health/anc-positive-pregnancy-experience/en/

33. Gureje O, Oladeji BD, Araya R, Montgomery AA, Kola L, Kirmayer L, Zelkowitz P, Groleau D, Kimayer L, Zelkowitz P, Groleau D. Expanding care for perinatal women with depression (EXPONATE): Study protocol for a randomized controlled trial of an intervention package for perinatal depression in primary care. BMC Psychiatry. 2015;15(1):136.

For peer review only - http://bmjopen.bmj.com/site/about/guidelines.xhtml

Table 1. Participants

Stage of pregnancy	India		Pakistan		Total number of women
	Method	Number of women	Method	Number of women	
First half of pregnancy (≤20 weeks)	2 FGD	16	2 FGD	26	42
Second half of pregnancy (>20 weeks)	3 FGD	17	2 FGD	24	41
Early postnatal stage (day 1-7)	3 FGD	22	10 interviews	10	32
Late postnatal stage (week 2-6)	1 FGD	5	10 interviews	10	15
**TOTAL**		**60**		**70**	**130**

For peer review only

Table 2: Women's understanding of health and ill-health in general

Health Sub-theme	Illustrative quotes	
**Diet**	Q1	"If a person is happy but not eating well, how can she be healthy? Diet is so important." (FGD 1, ANC, Pakistan)
	Q2	"Good health is good food." (Interview 6, PNC, Pakistan)
	Q3	"Health means to have balanced diet." (FGD 5, ANC, India)
**Weight**	Q4	"One should be strong and a bit bulky." (FGD 1, ANC, India)
	Q5	"Healthy women are those who are slim and have strength." (Interview 3, PNC, Pakistan)
**Energy**	Q6	"Good health is when there is no disease." (FGD 3, ANC, Pakistan)
	Q7	"Women should have the energy to do the chores, that means they are healthy." (FGD 4, ANC, Pakistan)
**Mental health**	Q8	"Being healthy means having no stress or worries and having a tension-free life." (FGD 5, ANC, India)
	Q9	"There is health of the mind and other body parts. If one body part is missing or is not healthy, one cannot be considered healthy. Similarly, if one is not mentally healthy, one is not able to do anything." (FGD 1, ANC, Pakistan)
	Q10	"Good health means being mentally, socially, emotionally and physically fit." (FGD 2, ANC, India)
**Social health**	Q11	"If there is peace in the home and in the mind, then it is good health." (Interview 3, PNC, Pakistan)
	Q12	"If there are fights in the home, this effects a woman's health negatively." (Interview 8, PNC, Pakistan)
	Q13	"The surrounding environment and family atmosphere directly affects [physical] health." (FGD 4, PNC, India)
**Ill-health Sub-themes**	**Illustrative quotes**	
**Physical symptoms**	Q14	"Ill-health means presence of disease." (FGD 4, ANC, India)
	Q15	"Those who are not healthy, their face shows it; they have yellowish complexion and have dark circles around the eyes." (FGD 1, ANC, Pakistan)
	Q16	"Ill-health is like flu, fever and sore throats." (Interview 5, PNC, Pakistan)
	Q17	"Poor health means having pain, discomfort, fever and body ache." (Interview 4, PNC, Pakistan)
	Q18	"Disease is for example HIV/AIDS, weak bones, weakness." (Interview 5, PNC, Pakistan)
**Mental ill-health**	Q19	"Ill-health is about tension; even if there is a very low level of stress or tension it does great damage to a person." (Interview 2, PNC, Pakistan)
	Q20	"Mental health affects physical health. If one is mentally disturbed, won't be able to do anything, even if you are physically fit, you will feel weak." (Interview 9, PNC, Pakistan)

For peer review only - http://bmjopen.bmj.com/site/about/guidelines.xhtml

Table 3: Women's understanding of health and ill-health during and after pregnancy.

Health Sub-theme	Illustrative quotes	
**Diet**	Q21	"In pregnancy, dietary care is more important to remain healthy, as there is a baby as well." (FGD 4, ANC, India)
	Q22	"One needs to be extra careful and eat more in pregnancy because it is the matter of two lives now." (Interview 2, PNC, Pakistan
**Medication**	Q23	"Good health in pregnancy is when you take your medicine regularly and completely." (Interview 4, PNC, Pakistan)
**Extra care is required during and after pregnancy for health**	Q24	"You have to take really good care of yourself in pregnancy. If you become weak, the complications increase, and the baby will also be weak." (Interview 4, PNC, Pakistan)
	Q25	"Pregnant woman must take a nap for one and a half hour to two hours during daytime and try to take more rest at night." (FGD 2, PNC, India)
	Q26	"The most important thing is this that baby is healthy and to have the proper medical care so that both baby and mother stay healthy." (Interview 7, PNC, Pakistan)
**Mental components**	Q27	"Women should not have any tension during pregnancy." (FDG 3, ANC, India)
**Social components**	Q28	"If family members are quarrelsome and abusive, it affects both the mother and baby's health." (FGD 2, ANC, India)
**Ill- Health Sub-theme**	**Illustrative quotes**	
**Physical symptoms**	Q29	"Ill-health is …. abdominal pain, leaking per vagina, vomiting, loss of appetite and dizziness." (FGD 2, ANC, India)
	Q30	"There are many symptoms that are dangerous, like if there is lack of fluids or baby around the baby is not growing as it should be, or if baby is suffering due to negligence." (Interview 5, PNC, Pakistan)
	Q31	"Bleeding or swelling is dangerous." (FGD 3, ANC, Pakistan)
**Mental components of health**	Q32	"Stress affects health in pregnancy, if there is any stress or tension one cannot eat properly. It also affects the baby's health and our own health too." (Interview 4, PNC, Pakistan)
**Social components of health**	Q33	"If family members are quarrelsome and abusive, it affects both the mother and baby's health." (Interview 7, PNC, Pakistan)
	Q34	"The husband comes home after drinking and beats the woman." (FDG 2, ANC, India)
	Q35	"If a husband spends his entire income on drinking, he comes home late and beats his pregnant wife." (FDG 4, ANC, India)
**Gender preference of the baby**	Q36	"In some families, male gender preference is a major cause of stress and tension for pregnant women." (FDG 1, ANC, India)
	Q37	"This time also I have given birth to a girl child [and] because of this the family atmosphere is not good. My family members are abusing me and because of this I am going through a lot of stress." (FDG 5, PNC, India)

For peer review only

Table 4: Women's views regarding whether healthcare providers should enquire regarding mental and social ill-health

Sub-theme	Illustrative quotes	
**Mental and social ill-health is not currently addressed**	Q38	"They do not ask about mental health or the family situation. They just ask about test reports, blood deficiency" (FGD 4, ANC, Pakistan)
	Q39	"They ask in detail about me, about my diet and medicines, they take a complete history and ask about all my previous deliveries, but they never ask about my family environment or mental health." (Interview 10, PNC, Pakistan)
	Q40	"They usually don't ask much, and we also do not talk much; they check, give medicine, or sometimes ask, does your husband smoke and is there any disease in the family?" (Interview 7, PNC, Pakistan)
**Mental and social ill-health should be assessed**	Q41	"Yes, they should ask us." (FGD 4, ANC, India)
	Q42	"They should ask such questions, but they do not. They should ask about family conflicts and talk about the solutions." (Interview 11, PNC, Pakistan).
	Q43	"If one has some grief, sharing with someone lessens the grief, though it may not solve the problem, but releases stress." (Interview 6, PNC, Pakistan)
**Education**	Q44	"Women who are uneducated or unaware might get offended by these questions, others will not mind." (FGD 2, ANC, Pakistan)
	Q45	"People who understand such issues will not react, but others will." (FGD 3, ANC, India)
**Stigma**	Q46	"Some women will not talk about their problems as they are afraid. They think that their husband will know about it if they talk to doctor and will be angry with them." (FGD 4, ANC, Pakistan)
	Q47	"I will feel ashamed to talk about my condition… I can tolerate until it is really necessary." (Interview 8, PNC, Pakistan)
	Q48	"Some women don't share. Some women keep everything inside." (Interview 11, PNC, Pakistan)
**Should not be assessed**	Q49	"Doctors should not ask these questions, no, they should not." (Interview 5, PNC, Pakistan)
	Q50	"No, doctor has no right to ask such questions." (Interview 6, PNC, Pakistan)
	Q51	"A doctor has many patients …Obviously, they don't have time for these details!" (FGD 4, ANC, Pakistan)
**Impact on family**	Q52	"If you ask about the family situation, our community is such that women will be afraid as to why these questions are being asked and they have a fear that in-laws will find out" (FGD 2, ANC, Pakistan)
	Q53	"If the doctor asks in front of our family, that will help change bad behaviours in the family" (FGD 3, ANC, Pakistan)
**Solutions**	Q54	"They should use questions in a way that are focused and clear." (FGD3, ANC, Pakistan)
	Q55	"Do you have any tension? How is your home environment? How is your husband and your family-in-laws behaviour with you?" (Interview 10, PNC, Pakistan)

For peer review only - http://bmjopen.bmj.com/site/about/guidelines.xhtml

For peer review only

For peer review only - http://bmjopen.bmj.com/site/about/guidelines.xhtml

LSTM
CENTRE FOR MATERNAL
AND NEWBORN HEALTH

Topic guides for women during and after pregnancy for their views and understanding of maternal health and ill-health

For peer review only

For peer review only - http://bmjopen.bmj.com/site/about/guidelines.xhtml

1. TOPIC GUIDE FOR PREGNANT AND POSTNATAL WOMEN

PROFILE OF WOMEN

Once participants have signed the consent form, ask them to answer these following questions. It will help analyse the qualitative data.

Study setting: ____

Age: ____

Parity: ____

Pregnant status / antenatal or postnatal and number of weeks: ______________________

FORMAT

- Introduce yourself and welcome the group.
- Explain that we are here to learn more about the groups' views on their general views regarding what they understand all types of ill-health to be during and after pregnancy.
- In addition, explain that we are keen to understand what the group in general think regarding questions concerning other types of ill-health that may be a risk factor to pregnancy and how best to approach and ask these questions routinely in their setting.
- Ensure everyone has provided information for the registration log sheet and has a participant information sheet.
- Obtain informed consent for each participant.
- Provide a brief introduction to the relevant ground rules for the key informant interview or focus group discussion:
  (1) The discussion will last a maximum of 1 hour
  (2) The content of the discussion will remain confidential and will not be used outside of the group except for the purposes of the research
  (3) Participants are also requested to keep the discussion confidential after the session
  (4) Participants' names will not be used when reporting the findings
  (5) Participants are encouraged to speak their mind and there are no right or wrong answers
  (6) Participants should not hesitate to disagree with someone else
  (7) But should not talk at once
  (8) The interview will be recorded using a small hand held voice recorder device.
- A voice recorder will be used only to ensure that none of the information from the FGD is lost, and to facilitate analysis of the data from the interviews.

For peer review only - http://bmjopen.bmj.com/site/about/guidelines.xhtml

MATERIALS

Topic guide, log, notepad, pens, voice recorders, batteries, information sheet, consent form, transport reimbursement and reimbursement form, refreshments, voice recorders.

FGD QUESTION GUIDE

Reminder to moderator: listen actively and always use probing questions to follow up and obtain further detail from the group (e.g. can you tell me more about that, can you give an example, why is that).

Introduction

We are very interested to hear about your general views regarding what you understand health and ill-health to be for women that you care for during and after pregnancy.
In addition, we are keen to understand how best to ask general questions concerning other types of ill-health that may be a risk factor to the pregnancy. These general questions will include how best to screen for psychological ill-health (depression) or social ill-health (domestic violence or substance misuse) during and after pregnancy.
We would like to understand your perceptions regarding the possible barriers or enabling factors to routinely screen women during and after pregnancy for all types of ill-health (physical, psychological and social) and whether you think women's general health needs are current being met in routine antenatal and postnatal care in your setting.

General questions to ask

1. What is health and what is ill-health during and after pregnancy?

We are interested to know about your general views of what health is and what ill-health in general is for women during and after their pregnancy.

- What do women in your setting consider to be 'good health'?
- What do women in your setting you consider to be 'poor health'?
- In relation to pregnancy: what would good health be and what would poor health be?
- If we consider poor health in pregnancy, what does this mean?
- What kinds of conditions or complaints do pregnant women tend to have?

- Is poor health different in pregnancy and after pregnancy? How?

- Probe: is health only related to physical health?

For peer review only - http://bmjopen.bmj.com/site/about/guidelines.xhtml

- Probe: does ill-health during and after pregnancy also include psychological, and social factors?
- Probe: In your opinion, do women have ill-health during and after their pregnancy? and if so what how does this bother them?

2. How best can a healthcare provider ask questions routinely regarding other forms of ill-health women experience during and after pregnancy?

We are keen to understand how best you think a healthcare provider can ask routinely regarding other forms of ill-health women experience during and after pregnancy, that may be a risk factor to the pregnancy, for example psychological ill-health (depression) or social ill-health (domestic violence or substance misuse)?

Can you tell us your general view and opinion on whether it is acceptable for healthcare providers to make enquiries regarding not just physical but also psychological and social ill-health during routine antenatal and postnatal care?

- Probe: What do you think about women being asked about their perception of their health in pregnancy?
- What kinds of questions do you think pregnant women should be asked to assess how they feel about their health?

- What do you think women would think regarding being asked about their mental well-being or social factors?
- Is it acceptable to be asked about this? Why? Why not?
- How do you think women should be asked these questions?

3. We would like to understand your general perceptions regarding the possible barriers or enabling factors to routinely screen women during and after pregnancy for all types of ill-health (physical, psychological and social).

- In your community, how is pregnancy perceived?
- Are pregnant women treated any differently? In what way?
- When women are pregnant do they expect to feel ill?
- What kinds of conditions or complaints are perceived as 'routine' during pregnancy?
- What conditions or complaints are not considered a 'routine' part of pregnancy?
- How do women deal with minor common discomforts s during pregnancy?

For peer review only - http://bmjopen.bmj.com/site/about/guidelines.xhtml

- How do women deal with those that are not considered a common part of pregnancy?

4. Do you think women's general health needs are current being met in routine antenatal and postnatal care in your setting?

We would like to understand more about whether the existing health care system in your general opinion, meets the health needs of women during and after pregnancy in your community?

Probe: To what extent do you think the health facility meets womens' needs during and after pregnancy? In your opinion does the health system in general provide good quality to women during and after pregnancy?

Probe: What do you think should be done to improve provision of services for women during and after pregnancy?

In summary:
Ask if participants would like to add further comments.

Bring the meeting to a close by summarizing the main points.

Do not forget to say thank you to the participants for their time and active participation.

For peer review only

For peer review only - http://bmjopen.bmj.com/site/about/guidelines.xhtml

COREQ (COnsolidated criteria for REporting Qualitative research) Checklist

A checklist of items that should be included in reports of qualitative research. You must report the page number in your manuscript where you consider each of the items listed in this checklist. If you have not included this information, either revise your manuscript accordingly before submitting or note N/A.

Topic	Item No.	Guide Questions/Description	Reported on Page No.
**Domain 1: Research team and reflexivity**			
Personal characteristics			
Interviewer/facilitator	1	Which author/s conducted the interview or focus group?	
Credentials	2	What were the researcher's credentials? E.g. PhD, MD	
Occupation	3	What was their occupation at the time of the study?	
Gender	4	Was the researcher male or female?	
Experience and training	5	What experience or training did the researcher have?	
Relationship with participants			
Relationship established	6	Was a relationship established prior to study commencement?	
Participant knowledge of the interviewer	7	What did the participants know about the researcher? e.g. personal goals, reasons for doing the research	
Interviewer characteristics	8	What characteristics were reported about the inter viewer/facilitator? e.g. Bias, assumptions, reasons and interests in the research topic	
**Domain 2: Study design**			
Theoretical framework			
Methodological orientation and Theory	9	What methodological orientation was stated to underpin the study? e.g. grounded theory, discourse analysis, ethnography, phenomenology, content analysis	
Participant selection			
Sampling	10	How were participants selected? e.g. purposive, convenience, consecutive, snowball	
Method of approach	11	How were participants approached? e.g. face-to-face, telephone, mail, email	
Sample size	12	How many participants were in the study?	
Non-participation	13	How many people refused to participate or dropped out? Reasons?	
Setting			
Setting of data collection	14	Where was the data collected? e.g. home, clinic, workplace	
Presence of non-participants	15	Was anyone else present besides the participants and researchers?	
Description of sample	16	What are the important characteristics of the sample? e.g. demographic data, date	
Data collection			
Interview guide	17	Were questions, prompts, guides provided by the authors? Was it pilot tested?	
Repeat interviews	18	Were repeat inter views carried out? If yes, how many?	
Audio/visual recording	19	Did the research use audio or visual recording to collect the data?	
Field notes	20	Were field notes made during and/or after the inter view or focus group?	
Duration	21	What was the duration of the inter views or focus group?	
Data saturation	22	Was data saturation discussed?	
Transcripts returned	23	Were transcripts returned to participants for comment and/or	

For peer review only

Topic	Item No.	Guide Questions/Description	Reported on Page No.
		correction?	
**Domain 3: analysis and findings**			
Data analysis			
Number of data coders	24	How many data coders coded the data?	
Description of the coding tree	25	Did authors provide a description of the coding tree?	
Derivation of themes	26	Were themes identified in advance or derived from the data?	
Software	27	What software, if applicable, was used to manage the data?	
Participant checking	28	Did participants provide feedback on the findings?	
Reporting			
Quotations presented	29	Were participant quotations presented to illustrate the themes/findings? Was each quotation identified? e.g. participant number	
Data and findings consistent	30	Was there consistency between the data presented and the findings?	
Clarity of major themes	31	Were major themes clearly presented in the findings?	
Clarity of minor themes	32	Is there a description of diverse cases or discussion of minor themes?	

Developed from: Tong A, Sainsbury P, Craig J. Consolidated criteria for reporting qualitative research (COREQ): a 32-item checklist for interviews and focus groups. *International Journal for Quality in Health Care*. 2007. Volume 19, Number 6: pp. 349 – 357

Once you have completed this checklist, please save a copy and upload it as part of your submission. DO NOT include this checklist as part of the main manuscript document. It must be uploaded as a separate file.

For peer review only

BMJ Open

“Good health means being mentally, socially, emotionally and physically fit”: Women’s understanding of health and ill-health during and after pregnancy in India and Pakistan: a qualitative study

Journal:	*BMJ Open*
Manuscript ID	bmjopen-2018-028760.R3
Article Type:	Research
Date Submitted by the Author:	26-Nov-2019
Complete List of Authors:	McCauley, Mary; Liverpool School of Tropical Medicine, Centre for Maternal and Newborn Health<br>Avais, Ayesha ; Child Advocacy International Pakistan<br>Agrawal, Ritu; Vardhman Mahavir Medical College and Safdarjung Hospital<br>Saleem, Shumaila; Child Advocacy International Pakistan<br>Zafar, S; Fazaia Medical College<br>van den Broek, Nynke; Liverpool School of Tropical Medicine, Centre for Maternal and Newborn Health
**Primary Subject Heading**:	Obstetrics and gynaecology
Secondary Subject Heading:	Evidence based practice, Global health
Keywords:	Maternal medicine < OBSTETRICS, Antenatal care, Postnatal care, Maternal morbidity, Understanding of health and ill-health

SCHOLARONE™
Manuscripts

"Good health means being mentally, socially, emotionally and physically fit": Women's understanding of health and ill-health during and after pregnancy in India and Pakistan: a qualitative study

Authors:

MCCAULEY, Mary[1]*

AVAIS, Ayesha Rasheeda[2]

AGRAWAL, Ritu [3]

SALEEM, Shumaila[2]

ZAFAR, Shamsa[4]

VAN DEN BROEK, Nynke[1]

[1]Centre for Maternal and Newborn Health, Liverpool School of Tropical Medicine, United Kingdom

[2]Child Advocacy International Pakistan, Islamabad, Pakistan

[3]Safdarjung Hospital, New Delhi, India

[4]Fazaia-Medical College, Air University, Islamabad, Pakistan

*Corresponding author: mary.mccauley@lstmed.ac.uk

Word count: 3769

Ethical Approval: The Liverpool School of Tropical Medicine, Liverpool, United Kingdom; Safdarjung Hospital, New Delhi and the National Bioethics Committee, Islamabad, Pakistan all granted ethical approval.

Disclosure of Funding Received: This study was funded by a Research Development Fund (RDF160301MMC11) from the Liverpool School of Tropical Medicine, Liverpool, United Kingdom.

Short Title: Women's views on health and ill-health during and after pregnancy in India and Pakistan.

Keywords: Health, ill-health, antenatal care, postnatal care, pregnancy, maternal morbidity.

For peer review only - http://bmjopen.bmj.com/site/about/guidelines.xhtml

For peer review only

Abstract

Objective

To explore what women consider health and ill-health to be, in general, and during and after pregnancy. Women's views on how to approach screening for mental ill-health and social morbidities were also explored.

Settings

Public hospitals in New Delhi, India and Islamabad, Pakistan.

Participants

130 women attending for routine antenatal or postnatal care at the study healthcare facilities.

Interventions

Data collection was conducted using focus group discussions and key informant interviews. Transcribed interviews were coded by topic and grouped into categories. Thematic framework analysis identified emerging themes.

Results

Women are aware that maternal health is multidimensional and linked to the health of the baby. Concepts of good health included: nutritious diet; ideal weight; absence of disease; and a supportive family environment. Ill-health consisted of physical symptoms and medical disease; stress/tension; domestic violence; and alcohol abuse in the family. Reported barriers to routine enquiry regarding mental and social ill-health included a small number of women's perceptions that these issues are 'personal'; that healthcare providers do not have the time and/or cannot provide further care, even if mental or social ill-health is disclosed.

Conclusions

Women have a good understanding of the comprehensive nature of health and ill-health during and after pregnancy. Women report that enquiry regarding mental and social ill-health is not part of routine maternity care, but most welcome such an assessment. Healthcare providers have a duty of care to deliver respectful care that meets the health needs of women in a comprehensive, integrated, holistic manner, including mental and social care. There is a need for further research to understand how to support healthcare providers to screen for all aspects of maternal morbidity (physical, mental and social); and for healthcare providers to

For peer review only

be enabled to provide support and evidence-based care and/or referral for women if any ill-health is disclosed.

Keywords

Health, ill-health, maternal health, women, antenatal care, postnatal care, qualitative research, perceptions.

Word count: 299

Strengths and limitations of this study

- We interviewed 130 women attending for routine antenatal or postnatal care in secondary level public hospitals in two different countries, India and Pakistan.
- We conducted both focus group discussions and key informant semi-structured interviews to explore a large number of women's own opinions.
- Similar proportions of women were interviewed at four different stages: (1) first half of pregnancy (≤20 weeks), (2) second half of pregnancy (>20 weeks), (3) early postnatal stage (day 1-7); or (4) late postnatal stage (week 2-6) after childbirth.
- We only interviewed women in urban settings and excluded women living in rural communities who may have alternative perspectives or different insights.

For peer review only

Introduction

All women have the right to the highest attainable standard of health and well-being including physical, mental and social aspects [1,2]. The Sustainable Development Goals (SDG) envision that that every woman in every setting has an equal chance to survive and thrive [3]. SDG 3 is to ensure healthy lives and promote well-being for all [3]. The Global Strategy strives for a world in which every mother can enjoy a wanted and healthy pregnancy and childbirth and that pregnancy is an opportunity for a woman to receive care to thrive [1]. Currently this is not the case for many women living in low-and middle- income countries (LMIC). As part of the need to develop innovative strategies to achieve SDG 3, there has been a renewed focus on maternal ill-health or maternal morbidity in terms of definition, measurement, and interventions; and especially for women living in LMIC [4-10]. The burden of disease of various aspects of health (physical, mental and social) during and after pregnancy and their impact on women and/or pregnancy outcomes are currently not well documented in a comprehensive, standardised approach [4,5]. It is suggested that although maternal ill-health occurs across all countries, cultures, religions, socioeconomic status, and ages, the burden of disease is higher in women living in LMIC with an estimated three out of four women suffering at least one form of maternal morbidity during or after their pregnancy [4,5]. However, there is currently lack of literature regarding the understanding of what women consider maternal morbidity or ill-health to be during and after pregnancy across many LMIC.

Maternal ill-health or morbidity is a complex and broad concept and can be understood and described in various ways depending on different perspectives [8-10]. For example, women, men, families, communities, healthcare providers and policy makers may have mixed views and perceptions on what maternal ill-health is and the effect or risk of maternal morbidity during and after pregnancy for the women and/or her baby [9]. Maternal morbidity has been defined as “any health condition that is attributed to or aggravated by pregnancy and childbirth which has a negative impact on the woman’s wellbeing” [8]. However, there is still a lack of understanding and lack of data regarding what women themselves consider ill-health to be (physical and/or mental and/or social) and their experiences (subjective measures) compared to clinical findings (objective measures) when assessed and defined as morbidity by a healthcare provider; and there is especially a lack of data from women living in LMIC [4,5,

For peer review only - http://bmjopen.bmj.com/site/about/guidelines.xhtml

9]. The WHO have recently highlighted the need for qualitative studies that explore women's lived experiences of ill-health and perceptions of their health and health needs during and after pregnancy [8-10]. In many high-income countries women's ideas, concerns, expectations and choices regarding their care during and after pregnancy are well considered. However, there is lack of data of women's voices and views regarding what they consider health and wellbeing to be during and after pregnancy in many LMIC. An understanding of women's perceptions and opinions is essential to inform how healthcare providers can better deliver best care possible within existing healthcare systems in LMIC settings.

This study therefore sought to explore what women attending for routine antenatal care (ANC) or postnatal care (PNC) at healthcare facilities in India and Pakistan consider health and ill-health to be in general, and, what they consider health and ill-health to be, during and after pregnancy. As a component of maternal ill-health, women's views on how best to approach aspects of mental ill-health (such as depression and/or anxiety) and social ill-health (such as exposure to violence and/or substance misuse), were assessed.

Materials

Study design

Experienced female researchers (AA, RA, SS) interviewed a total of 130 women. Data collection was conducted using focus group discussions (FGD) to interview 60 women attending for routine ANC or PNC in the outpatient clinics at a large public hospital in New Delhi, India. FDGs and key informant interviews were used to interview 70 women attending for routine ANC or PNC in the outpatient clinics at a large public hospital in Islamabad, Pakistan. The sampling frame included (1) women who were pregnant or who have given birth within the past six weeks and (2) women who above the legal age of consent. Purposive sampling was used to approach women attending each study setting healthcare facility for routine ANC or PNC care. Women were only included once in the study. All interviews and FGDs were held in quiet locations away from the clinical areas at the relevant hospitals.

Participants and settings

All women attending for routine ANC or PNC in the study healthcare facilities were invited to participate. The study settings were two large public urban hospitals serving women with low-

For peer review only - http://bmjopen.bmj.com/site/about/guidelines.xhtml

lower middle socio-economic status; and with varied education levels [4]. Similar proportions of women were included from four different stages of pregnancy and FGDs included women within the same assessment stage of pregnancy (first half of pregnancy (≤20 weeks); second half of pregnancy (>20 weeks); early postnatal stage (day 1-7); or late postnatal stage (week 2-6). Women were purposively selected and recruited sequentially until the number of anticipated FGD and key informant interviews was reached, and data saturation was achieved.

Topic guide

A topic guide was developed to guide the interviews and was piloted in the United Kingdom with clinical researchers from LMIC (Supplementary document 1). The topic guide was then translated into the local study setting language, back-translation was conducted, and the topic guide was further piloted in each study setting. As a result, the topic guide was refined; for example, some wording was changed in the local language and the introduction was amended to ensure that the women were aware that we sought to assess their general views and not their own personal experiences (if any) of different types of ill-health. The topic guide served as a flexible tool to facilitate the facilitators in obtaining the women's opinions whilst ensuring that the interview remained on topic. The topic guide also acted as a cue to ask more probing questions to further understand women' beliefs and perceptions. The topic guide included three main subject areas:

1) perception and understanding of health and ill-health in general;

2) perception and understanding of health and ill-health during and after pregnancy;

3) views on enquiry regarding mental and social ill-health during and after pregnancy; and suggestions on how best to approach all components of health.

Data Collection

Prior to interview, all eligible women were approached and given verbal and written information regarding the study including a brief overview of the research aims and interview questions. Appointments for FGD or key informant interviews were then scheduled for a later time convenient for the women. The FGD and key informant interviews were conducted in the local language (India-Hindi and Pakistan-Urdu) by the local female trained and experienced researchers (AA, RA, SS). On average the FGDs lasted 60-90 minutes, and the key

For peer review only - http://bmjopen.bmj.com/site/about/guidelines.xhtml

informant interviews lasted 30 minutes. All FGD and key informant interviews were conducted face-to-face, recorded on a digital recording device and transcribed in the local language upon completion by the local researchers (AA, RA, SS). All efforts were made to emphasise confidentiality for interviews to ensure women were confident in giving honest answers and member checking was used to assure correct interpretation. Interviewing women from different stages of pregnancy, different backgrounds, and different countries broadened the scope of the topic and enabled transferability to be achieved to an extent.

Data analysis

In each study setting, transcribed interviews were translated into English and initially manually open coded following data familiarisation (reading and re-reading of the transcripts several times) by the local researchers (RA in India; AA and SS in Pakistan). In a sub-sample of cases, and prior to translation, participants were asked to review the coded transcripts to verify the accuracy and appropriateness of the applied code. Using thematic analysis, codes were identified from the transcripts and grouped into categories independently by the local researchers, enabling the first abstraction of data in each setting [11]. Thematic framework analysis of the categories was then undertaken. The separate results were then brought together and refined to agree on the key themes. All of the coded data was reviewed by other members of the research team (MMc in India and SZ in Pakistan) to ensure consistency and to check for interrater reliability. In addition, all members of the research team reviewed, and critiqued emerging themes and sub-themes and any discrepancies were discussed, and consensus agreed. The reporting of the results adheres to the COREQ guidelines [12].

Patient and Public Involvement

No patient involved.

Ethics

The Liverpool School of Tropical Medicine, Liverpool, United Kingdom, granted full ethical approval (LSTM 16-054RS). Ethical approval was also obtained from the Research and Ethics Committee, Vardhman Mahavir Medical College and Safdarjung Hospital, New Delhi, India (ICE/SJH/VMMC/Project/October-2016/688); and the National Bioethics Committee,

For peer review only

For peer review only - http://bmjopen.bmj.com/site/about/guidelines.xhtml

Islamabad, Pakistan (No.4-87/16/NBC-159-Addendum/RDC/3634). Written informed consent was obtained from each woman who participated in the study.

Results

A total of 130 women were interviewed (**Table 1**). Three women invited to participate declined due to time constraints. A total of 83 women were pregnant at the time of interview and 47 had given birth with the past six weeks. Most women were either in their first pregnancy or had one previous pregnancy. A minority of women had more than two previous pregnancies. The age range was from 20-36 with a mean of 26 years old. There were no distinct differences in emerging themes or sub-themes from women from India or Pakistan. Therefore, all quotes illustrating the results are combined for both countries and displayed in tables and linked to the narrative text.

What is health?

Women perceived "good health" in general as having a good diet; having an ideal weight; absence of physical disease; absence of stress and tension; and a supportive family environment (Table 2). Most women expressed that the ability to have a good diet would result in good health (Table 2, Q1-Q3). Weight was mentioned in India and Pakistan, however, some women's perception of this differed (Table 2, Q4-Q5). In both settings, women reported that health meant lack of disease and good energy levels (Table 2, Q6-Q7). Most women reported that health was not just physical health but includes mental health (Table 2, Q8-Q10). Most women highlighted that a supportive environment with family were important for good health (Table 2, Q11-Q13).

What is ill-health?

The emerging themes related to perceived ill-health included; physical symptoms and disease; tension; and an abusive and stressful home environment. Many women expressed that ill-health was "pain, flu, fever, sore throat, weakness, body ache, headache, and swelling" (Table 2, Q14-Q17). Ill-health was also described in terms of disease, for example HIV/AIDS (Table 2, Q18). Mental ill-health was often considered a health concern and stress and tension was reported by many women as a component of or factor leading to mental ill-health (Table 2, Q19-Q20).

For peer review only - http://bmjopen.bmj.com/site/about/guidelines.xhtml

What is health during and after pregnancy?

During and after pregnancy a nutritious diet, medication, rest, extra care, and a peaceful home environment were mentioned as important. Most women reported that diet is even more important when a woman is pregnant (Table 3, Q21-Q22). Many women mentioned that pregnant women needed medication and more care from the family and healthcare providers (Table 3, Q23-Q25). Many women understood that their health was "lack of tension" and that their (or ill-health) was linked to that of their baby (Table 3, Q26-Q28).

What is ill-health during and after pregnancy?

Perceived ill-health during and after pregnancy included the perception that pregnancy is a "disease", and that there was "no health in pregnancy", as all "the energy goes to the baby and the mother becomes weaker". Women also considered some physical symptoms and discomfort as "normal" during pregnancy. This included nausea and vomiting, back pain and difficulty sleeping. However, many women reported that ill-health during and after pregnancy was "vomiting, high blood pressure, bleeding, swelling, abdominal pain" (Table 3, Q29). Many women were aware that these symptoms could be dangerous (Table 3, Q30-Q31). Stress and tension were considered important components of ill-health by most women (Table 3, Q32). An abusive family environment was highlighted as examples of social ill-health in women during and after pregnancy (Table 3, Q33-Q35). There was some discussion regarding gender preference, and it was suggested by a minority of women that the gender of a baby created stress and tension for women (Table 3, Q36-Q37).

Should healthcare providers ask about mental and social ill-health?

An emerging theme regarding what women consider health and ill-health to be, was the importance of mental and social health and well-being. Women described mental ill-health such as "depression", "weeping", stress", "tension", "grief", "loss of appetite" and "unhappiness". Women reported social concerns such as "fights in the home", "domestic violence", "poor family environment", "quarrels and verbal abuse", "family or husband alcohol misuse", and an "expectation of a male child". Women were therefore asked questions regarding how best to approach and address the concepts of mental and social ill-

For peer review only - http://bmjopen.bmj.com/site/about/guidelines.xhtml

health for women during and after pregnancy. Most women reported that healthcare providers ask pregnant women questions regarding physical ill-health only (Table 4, Q38-Q40). Most women felt that there was a lack of enquiry from healthcare providers regarding mental and social ill-health during and after pregnancy. Most women felt that healthcare providers could and should ask women about their mental and social health (Table 4, Q41-Q43). Many women said that it would be beneficial to speak to their healthcare provider if they were feeling "stressed" and "under tension" as the healthcare provider might be able to provide general support and advice. However, a few women explained these types of questions might be considered unacceptable, because of lack of education and understanding among some women (Table 4, Q44-Q45). There was some concern that some women would prefer not to talk about their mental and social ill-health as they may feel afraid, ashamed or uncomfortable (Table 4, Q46-Q48). A small minority of women in Pakistan felt it was not the role of the healthcare provider to ask questions regarding mental and/or social health, as this was their "personal life". Women discussed the repercussions from husbands and family if domestic violence during and after pregnancy would be disclosed by a woman to a healthcare provider (Table 4, Q49-Q51). There were concerns that a woman would not be allowed to access care again. There was a lot of discussion about this with sometimes conflicting opinions; ranging from it not being right for a woman to disclose domestic violence, or mental ill-health such as stress and tension; to a consideration that healthcare providers should ask and would provide help. A few women said that if they themselves were asked questions regarding depression or domestic violence, they would seek care from another healthcare provider to avoid these questions. Many women recognised that there were existing barriers for healthcare providers which made it difficult for them to ask women questions regarding mental and social ill-health during and after pregnancy (Table 4, Q52-Q53). Such barriers included the perceived heavy workload of healthcare providers and lack of time to explore mental and/or social concerns (Table 4, Q51). Other women explained that "they can ask, but what can they do?" and that "nothing can be done" to help. Some women did offer suggestions of how healthcare providers could best ask questions regarding mental and social ill-health during and after pregnancy. A more empathic approach to women were suggested including politeness and friendliness were suggested. Confidentiality was highlighted as important by many women. Some women suggested that healthcare providers need not go into such detailed questions but keep any questions simple (Table 4, Q54-Q55).

For peer review only - http://bmjopen.bmj.com/site/about/guidelines.xhtml

Discussion

Statement of principal findings

Most women who took part in this study had clear opinions and had a good understanding that health includes more than physical components and that the health of the mother was linked to that of the baby. Good diet, healthy weight, rest and a supportive home environment contributed to good health; whereas physical disease symptoms, complications of pregnancy, as well as stress, tension and an abusive family environment contributed to ill-health. Women were aware of physical common discomforts and of more severe complications and dangers signs, as ill-health related to pregnancy. Women considered mental and social ill-health as important but reported that healthcare providers do not currently ask women how they are feeling or ask after their mental nor social health and wellbeing . However, most women would be happy if healthcare providers did so. A minority of women considered mental and social health concerns as "personal" and reported that there may be barriers to asking such questions.

Strengths of the study

To the best of our knowledge, this is the first study from two LMIC settings (India and Pakistan) to obtain women's own opinions on what they might consider health and ill-health to be in general and during and after pregnancy. A range of women from four different stages of pregnancy were interviewed resulting in a wide spectrum of responses. Most women approached welcomed the discussion surrounding health, and ill-health during and after pregnancy, and were keen to contribute to solutions in their settings regarding healthcare providers roles in routine enquiry regarding other components of health including mental and social aspects, and not just physical health.

Limitations of the study

This study population included women attending for routine ANC or PNC in public secondary level healthcare facilities in urban settings and excludes other women who may have alternative perspectives or different insights. There is a need to assess the views of women

For peer review only - http://bmjopen.bmj.com/site/about/guidelines.xhtml

in other LMIC and including community and rural settings, as these women may have different perceptions and understandings.

How does this study relate to other literature?

There is lack of literature regarding the understanding of what women consider health and ill-health to be during and after pregnancy in India and Pakistan. There is also debate that the current definition of health [2] is out-dated and needs to be re-formulated to consider health in a context of functionality, capacity, adaptability and the ability to perform activities of daily living despite having a disease or disability [13]. There have been various proposals to develop the definition of health using the concept of "health, as the ability to adapt and to self-manage" but with a continued emphasis on the importance of the three domains of health: physical, mental, and social [13]. Aspects of physical ill-health in women during and after pregnancy in LMIC settings are well documented and there are many recommendations and interventions developed to diagnose and manage these conditions; for example, anaemia, haemorrhage, hypertensive disorders of pregnancy, infection, and complications of obstructed labour [14-18]. With regards to mental and social ill-health in women during and after pregnancy in LMIC, there is emerging data to demonstrate that depression and domestic violence represent a large burden of ill-health and have a negative impact on a woman's health and well-being [19-22]. However, to date, clinical practice in low resource settings rarely takes mental or social ill-health into account when assessing a woman's health needs during and after pregnancy [23-24]. However, this study demonstrates that most women would welcome such an assessment but report that their healthcare providers do not current ask; and even if they did, some women report that "nothing that can be done". This may be due to a shortage of human resources but also because of healthcare providers' perceptions that these issues are not "ill-health" per se and because of perceived cultural sensitivities related to mental ill-health and -social concerns [4,23-26].

It is well recognised that maternal physical, mental and social ill-health is linked to poor fetal and newborn health outcomes and women in our study demonstrated an understanding of this important association [27-30]. The detrimental impact of mental and social morbidity on the overall health and well-being of mothers (and their babies) during and after pregnancy

For peer review only - http://bmjopen.bmj.com/site/about/guidelines.xhtml

has resulted in public health policy in high-income countries such as the United Kingdom (UK), where enquiry regarding of mental ill-health (depression, anxiety) and social circumstances (exposure to domestic violence and/or substance misuse), is routinely conducted during and after pregnancy [31]. The WHO have produced clinical and policy guidelines on how to comprehensively care for women during and after pregnancy including identification, safety assessment and planning, communication and clinical skills, documentation and provision of referral pathways for mental and social ill-health [32]. However, the feasibility of implementation and acceptability of this guidance in countries, such as India and Pakistan are currently uncertain. There is debate as to the cadre of healthcare providers most suitable to undertake routine enquiry regarding for and/or manage mental and social ill-health in LMIC [23,24,33]. In many high-income countries, specially trained midwives routinely assess, screen, support and provide further referral for women with mental and social morbidity [31]. This study shows that most women are keen to be asked questions regarding their mental and social well-being during and after pregnancy, as part of routine maternity care. However, a minority of women do not wish to be asked.

Currently, there is good coverage and uptake of antenatal care, but content needs to be adapted to ensure good quality comprehensive care that is respectful, integrated and delivers physical, mental and social care, with agreed country specific additional content. Although, mental and social ill-health is clearly a real matter of concern for women, this study was not designed to explore this in further detail. Further research is needed to explain in more depth what these problems are, how they can be addressed, and by whom. It is important that healthcare providers are supported to provide good quality care for women and that this goes beyond simply the physical aspects of health and is inclusive of mental and social well-being.

Conclusion

Women reported that health and ill-health is not just physical but also includes mental and social components. Maternal ill-health is common among women attending for antenatal and postnatal care [4]. Women are increasingly accessing care during and after pregnancy and there is a window of opportunity to adapt and amend available care packages to include comprehensive enquiry regarding and, where needed, support for mental and social ill-health

For peer review only - http://bmjopen.bmj.com/site/about/guidelines.xhtml

[4, 10]. This study demonstrates that most women are keen to be asked questions regarding their mental and social well-being and provides simple suggestions that healthcare providers can use to improve the enquiry regarding mental and social ill-health among women during and after pregnancy. There is a need for further research to help develop interventions and deliver maternity care in a way that meets the health needs of women in a comprehensive and holistic manner, including mental and social components [4, 5].

Abbreviations

ANC: antenatal, **FGD**: Focus group discussion, **LMIC**: Low and middle-income country, **LSTM**: Liverpool School of Tropical Medicine, **SDG**: Sustainable Development Goals, **PNC:** postnatal, **UK**: United Kingdom, **WHO**: World Health Organization

Declarations

Ethics approval and consent to participate

The Liverpool School of Tropical Medicine, Liverpool, United Kingdom, granted full ethical approval (LSTM 16-054RS). Ethical approval was also obtained from the Research and Ethics Committee, Vardhman Mahavir Medical College and Safdarjung Hospital, New Delhi, India (ICE/SJH/VMMC/Project/October-2016/688); and the National Bioethics Committee, Islamabad, Pakistan (No.4-87/16/NBC-159-Addendum/RDC/3634). Written informed consent was obtained from each woman who participated in the study.

Consent for publication

Written informed consent was obtained from all women in the study, including audio-recording and the use of excerpts in publications and reports.

Availability of data and material

The datasets used and analysed during the current study are available from the corresponding author on reasonable request.

Competing interests

The authors declare that they have no competing interests.

Funding

Disclosure of funding received: This study was funded by the Liverpool School of Tropical Medicine Research Development Fund (RDF160301MMC11).

For peer review only - http://bmjopen.bmj.com/site/about/guidelines.xhtml

Author's contributions

MMC conceived the study idea, design and developed the topic guide. SZ co-ordinated and supervised the research activities in Pakistan. AA, SS and RA conducted the interviews, transcription, translations and data analysis. MMc co-ordinated and supervised the overall research activities, interpreted and presented the results and wrote the manuscript. NvdB has reviewed the data, interpreted the results and contributed to the manuscript. All authors have read, edited and approved the final manuscript for submission.

Acknowledgements

The authors would like to thank Professor Pratima Mittal, Professor Jyotsna Suri and Dr Mansi for their support with ethics applications and data collection in India. A huge thank you to all the women who participated in this study.

For peer review only

References

1. United Nations. Global Strategy for Women's, Children's and Adolescents' Health, 2016-2030. New York: United Nations; 2015. http://www.who.int/life-course/partners/global-strategy/en/
2. World Health Organization. Preamble to the Constitution of the World Health Organization as adopted by the International Health Conference, New York, 19-22 June 1946. http://www.who.int/about/definition/en/print.html
3. United Nations. Sustainable Development Goals. http://www.un.org/sustainabledevelopment/sustainable-development-goals/
4. McCauley M, Madaj B, White SA, Dickinson F, Bar-Zev S, Aminu M, Godia P, Mittal P, Zafar S, van den Broek N. Burden of physical, psychological and social ill-health during and after pregnancy among women in India, Pakistan, Kenya and Malawi. BMJ Glob Health. 2018 May 3;3(3):e000625. doi: 10.1136/bmjgh-2017-000625. PubMed PMID: 29736274; PubMed Central PMCID: PMC5935159.
5. Zafar S, Jean-Baptiste R, Rahman A, Neilson JP, van den Broek N. Non-Life Threatening Maternal Morbidity: Cross Sectional Surveys from Malawi and Pakistan. PLoS ONE. 2015;10(9):e0138026.
6. Say L, Souza JP, Pattinson RC, for the WHO working group on Maternal Mortality and Morbidity classifications. Maternal near miss - towards a standard tool for monitoring quality of maternal health care. Best Pract Res Clin Obstet Gynaecol. 2009;23(3):287-96.
7. Chou D, Tunçalp Ö, Firoz T, Barreix M, Filippi V, von Dadelszen P, van den Broek N, Cecatti JG, Say L. Constructing maternal morbidity – towards a standard tool to measure and monitor maternal health beyond mortality. BMC Pregnancy Childbirth. 2016;16:45.
8. Barreix M , Barbour K, McCaw-Binns A, Chou D, Petzold M, Gichuhi GN, Gadama L, Taulo F, Tunçalp Ö, Say L (2018). Standardizing the measurement of maternal morbidity: Pilot study results. Int J Gynecol Obstet, 141: 10-19.
9. Say L, Chou D. (2018), Maternal morbidity: Time for reflection, recognition, and action. Int J Gynecol Obstet, 141: 1-3.
10. Filippi V, Chou D, Barreix M, Say L. (2018), A new conceptual framework for maternal morbidity. Int J Gynecol Obstet, 141: 4-9.

For peer review only - http://bmjopen.bmj.com/site/about/guidelines.xhtml

11. Firoz T, Chou D, von Dadelszen P, Agrawal P, Vanderkruik R, Tunçalp O, Magee LA, van den Broek N, Say L. Measuring maternal health: focus on maternal morbidity. Bull World Health Organ. 2013;91(10):794-6.
12. Braun V, Clarke V. Using thematic analysis in psychology. Qual Res Psychol. 2006;3(2):77-101.
13. Equator Network. Consolidated criteria for reporting qualitative research (COREQ): a 32-item checklist for interviews and focus groups. http://www.equator-network.org/reporting-guidelines/coreq/
14. Huber M, Knottnerus JA, Green L, van der Horst H, Jadad AR, Kromhout D, Leonard B, Lorig K, Loureiro MI, van der Meer JW, et al. How should we define health? BMJ. 2011;343:d416
15. World Health Organization. The WHO Application of ICD-10 to deaths during pregnancy, childbirth and the puerperium: ICD-MM. Geneva: World Health Organization; 2012. http://www.who.int/reproductivehealth/publications/monitoring/9789241548458/en/
16. Abalos E, Cuesta C, Grosso AL, Chou D, Say L. Global and regional estimates of pre-eclampsia and eclampsia: a systematic review. Eur J Obstet Gynecol Reprod Biol. 2013;170(1):1-7
17. Calvert C, Thomas SL, Ronsmans C, Wagner KS, Adler AJ, Filippi V. Identifying regional variation in the prevalence of postpartum haemorrhage: a systematic review and meta-analysis. PLoS ONE. 2012;7(7):e41114.
18. World Health Organization. Haemoglobin concentrations for the diagnosis of anaemia and assessment of severity. Geneva: World Health Organization; 2011. http://www.who.int/vmnis/indicators/haemoglobin.pdf
19. Velu PP, Gravett CA, Roberts TK, Wagner TA, Zhang JSF, Rubens CE, Gravett MG, Campbell H, Rudan I. Epidemiology and aetiology of maternal bacterial and viral infections in low- and middle-income countries. J Glob Health. 2011;1(2):171-88.
20. World Health Organization. Maternal Mental Health; 2017. http://www.who.int/mental_health/maternal-child/maternal_mental_health/en/
21. Vos T, Barber RM, Bell B, Bertozzi-Villa A, Biryukov S, Bollger I and Global Burden of Disease Study 2013 Collaborators.. Global, regional, and national incidence, prevalence, and years lived with disability for 301 acute and chronic diseases and injuries in 188

For peer review only

For peer review only - http://bmjopen.bmj.com/site/about/guidelines.xhtml

countries, 1990-2013: a systematic analysis for the Global Burden of Disease Study 2013. Lancet. 2015;386(9995):743-800.

22. Shrivastava SR, Shrivastava PS, Ramasamy J. Antenatal and postnatal depression: a public health perspective. J Neuros Rural Prac. 2015;6(1):116-9.
23. McCauley M, Brown A, Ofosu B, van den Broek N. “I just wish it becomes part of routine care?”:Healthcare providers’ knowledge, attitudes and perceptions of screening for maternal mental health during and after pregnancy. BMC Psychiatry 2019; (19): 279
24. McCauley M, Head J, Lambert J, Zafar S, van den Broek, N. “Keeping family matters behind closed doors": Healthcare providers' perceptions and experiences of identifying and managing domestic violence during and after pregnancy. BMC Pregnancy Childbirth. 2017;(17)1:318.
25. World Health Organization. Understanding and addressing violence against women. Geneva: World Health Organization; 2012. http://apps.who.int/iris/bitstream/10665/77432/1/WHO_RHR_12.36_eng.pdf
26. World Health Organization. Global and regional estimates of violence against women: prevalence and health effects of intimate partner violence and non-partner sexual violence Geneva: World Health Organization; 2013. http://apps.who.int/iris/bitstream/10665/85239/1/9789241564625_eng.pdf?ua=1
27. World Health Organization. Framing the health workforce agenda for the Sustainable Development Goals: Biennium report 2016-2017 WHO Health Workforce. Geneva: World Health Organization; 2017 http://www.who.int/hrh/BienniumReportRevised2017.pdf
28. Gelaye B, Rondon MB, Araya R, Williams MA. Epidemiology of maternal depression, risk factors, and child outcomes in low-income and middle-income countries. Lancet Psychiatry. 2016;3(10):973.
29. Zakar R, Nasrullah M, Zakar MZ, Ali H. The association of intimate partner violence with unintended pregnancy and pregnancy loss in Pakistan. Int J Gynecol Obstet. 2016;133:26-31.
30. Satyanarayana VA, Lukose A, Srinivasan K. Maternal mental health in pregnancy and child behavior. Indian J Psychiatry. 2011;53(4):351.

For peer review only

31. National Institute for Health and Care Excellence. Antenatal and postnatal mental health: clinical management and service guidance. London: NICE; 2015. https://www.nice.org.uk/guidance/cg192

32. World Health Organization. WHO recommendations on antenatal care for a positive pregnancy experience. Geneva: World Health Organization; 2016. http://www.who.int/reproductivehealth/publications/maternal_perinatal_health/anc-positive-pregnancy-experience/en/

33. Gureje O, Oladeji BD, Araya R, Montgomery AA, Kola L, Kirmayer L, Zelkowitz P, Groleau D, Kimayer L, Zelkowitz P, Groleau D. Expanding care for perinatal women with depression (EXPONATE): Study protocol for a randomized controlled trial of an intervention package for perinatal depression in primary care. BMC Psychiatry. 2015;15(1):136.

For peer review only

For peer review only - http://bmjopen.bmj.com/site/about/guidelines.xhtml

Table 1. Participants

Stage of pregnancy	India		Pakistan		Total number of women
	Method	Number of women	Method	Number of women	
First half of pregnancy (≤20 weeks)	2 FGD	16	2 FGD	26	42
Second half of pregnancy (>20 weeks)	3 FGD	17	2 FGD	24	41
Early postnatal stage (day 1-7)	3 FGD	22	10 interviews	10	32
Late postnatal stage (week 2-6)	1 FGD	5	10 interviews	10	15
**TOTAL**		**60**		**70**	**130**

For peer review only

Table 2: Women's understanding of health and ill-health in general

Health Sub-theme	Illustrative quotes	
**Diet**	Q1	"If a person is happy but not eating well, how can she be healthy? Diet is so important." (FGD 1, ANC, Pakistan)
	Q2	"Good health is good food." (Interview 6, PNC, Pakistan)
	Q3	"Health means to have balanced diet." (FGD 5, ANC, India)
**Weight**	Q4	"One should be strong and a bit bulky." (FGD 1, ANC, India)
	Q5	"Healthy women are those who are slim and have strength." (Interview 3, PNC, Pakistan)
**Energy**	Q6	"Good health is when there is no disease." (FGD 3, ANC, Pakistan)
	Q7	"Women should have the energy to do the chores, that means they are healthy." (FGD 4, ANC, Pakistan)
**Mental health**	Q8	"Being healthy means having no stress or worries and having a tension-free life." (FGD 5, ANC, India)
	Q9	"There is health of the mind and other body parts. If one body part is missing or is not healthy, one cannot be considered healthy. Similarly, if one is not mentally healthy, one is not able to do anything." (FGD 1, ANC, Pakistan)
	Q10	"Good health means being mentally, socially, emotionally and physically fit." (FGD 2, ANC, India)
**Social health**	Q11	"If there is peace in the home and in the mind, then it is good health." (Interview 3, PNC, Pakistan)
	Q12	"If there are fights in the home, this effects a woman's health negatively." (Interview 8, PNC, Pakistan)
	Q13	"The surrounding environment and family atmosphere directly affects [physical] health." (FGD 4, PNC, India)
**Ill-health Sub-themes**	**Illustrative quotes**	
**Physical symptoms**	Q14	"Ill-health means presence of disease." (FGD 4, ANC, India)
	Q15	"Those who are not healthy, their face shows it; they have yellowish complexion and have dark circles around the eyes." (FGD 1, ANC, Pakistan)
	Q16	"Ill-health is like flu, fever and sore throats." (Interview 5, PNC, Pakistan)
	Q17	"Poor health means having pain, discomfort, fever and body ache." (Interview 4, PNC, Pakistan)
	Q18	"Disease is for example HIV/AIDS, weak bones, weakness." (Interview 5, PNC, Pakistan)
**Mental ill-health**	Q19	"Ill-health is about tension; even if there is a very low level of stress or tension it does great damage to a person." (Interview 2, PNC, Pakistan)
	Q20	"Mental health affects physical health. If one is mentally disturbed, won't be able to do anything, even if you are physically fit, you will feel weak." (Interview 9, PNC, Pakistan)

For peer review only

Table 3: Women's understanding of health and ill-health during and after pregnancy.

Health Sub-theme	Illustrative quotes	
**Diet**	Q21	"In pregnancy, dietary care is more important to remain healthy, as there is a baby as well." (FGD 4, ANC, India)
	Q22	"One needs to be extra careful and eat more in pregnancy because it is the matter of two lives now." (Interview 2, PNC, Pakistan)
**Medication**	Q23	"Good health in pregnancy is when you take your medicine regularly and completely." (Interview 4, PNC, Pakistan)
**Extra care is required during and after pregnancy for health**	Q24	"You have to take really good care of yourself in pregnancy. If you become weak, the complications increase, and the baby will also be weak." (Interview 4, PNC, Pakistan)
	Q25	"Pregnant woman must take a nap for one and a half hour to two hours during daytime and try to take more rest at night." (FGD 2, PNC, India)
	Q26	"The most important thing is this that baby is healthy and to have the proper medical care so that both baby and mother stay healthy." (Interview 7, PNC, Pakistan)
**Mental components**	Q27	"Women should not have any tension during pregnancy." (FDG 3, ANC, India)
**Social components**	Q28	"If family members are quarrelsome and abusive, it affects both the mother and baby's health." (FGD 2, ANC, India)
**Ill- Health Sub-theme**	**Illustrative quotes**	
**Physical symptoms**	Q29	"Ill-health is …. abdominal pain, leaking per vagina, vomiting, loss of appetite and dizziness." (FGD 2, ANC, India)
	Q30	"There are many symptoms that are dangerous, like if there is lack of fluids or baby around the baby is not growing as it should be, or if baby is suffering due to negligence." (Interview 5, PNC, Pakistan)
	Q31	"Bleeding or swelling is dangerous." (FGD 3, ANC, Pakistan)
**Mental components of health**	Q32	"Stress affects health in pregnancy, if there is any stress or tension one cannot eat properly. It also affects the baby's health and our own health too." (Interview 4, PNC, Pakistan)
**Social components of health**	Q33	"If family members are quarrelsome and abusive, it affects both the mother and baby's health." (Interview 7, PNC, Pakistan)
	Q34	"The husband comes home after drinking and beats the woman." (FDG 2, ANC, India)
	Q35	"If a husband spends his entire income on drinking, he comes home late and beats his pregnant wife." (FDG 4, ANC, India)
**Gender preference of the baby**	Q36	"In some families, male gender preference is a major cause of stress and tension for pregnant women." (FDG 1, ANC, India)
	Q37	"This time also I have given birth to a girl child [and] because of this the family atmosphere is not good. My family members are abusing me and because of this I am going through a lot of stress." (FDG 5, PNC, India)

For peer review only

Table 4: Women's views regarding whether healthcare providers should enquire regarding mental and social ill-health

Sub-theme	Illustrative quotes	
**Mental and social ill-health is not currently addressed**	Q38	"They do not ask about mental health or the family situation. They just ask about test reports, blood deficiency" (FGD 4, ANC, Pakistan)
	Q39	"They ask in detail about me, about my diet and medicines, they take a complete history and ask about all my previous deliveries, but they never ask about my family environment or mental health." (Interview 10, PNC, Pakistan)
	Q40	"They usually don't ask much, and we also do not talk much; they check, give medicine, or sometimes ask, does your husband smoke and is there any disease in the family?" (Interview 7, PNC, Pakistan)
**Mental and social ill-health should be assessed**	Q41	"Yes, they should ask us." (FGD 4, ANC, India)
	Q42	"They should ask such questions, but they do not. They should ask about family conflicts and talk about the solutions." (Interview 11, PNC, Pakistan).
	Q43	"If one has some grief, sharing with someone lessens the grief, though it may not solve the problem, but releases stress." (Interview 6, PNC, Pakistan)
**Education**	Q44	"Women who are uneducated or unaware might get offended by these questions, others will not mind." (FGD 2, ANC, Pakistan)
	Q45	"People who understand such issues will not react, but others will." (FGD 3, ANC, India)
**Stigma**	Q46	"Some women will not talk about their problems as they are afraid. They think that their husband will know about it if they talk to doctor and will be angry with them." (FGD 4, ANC, Pakistan)
	Q47	"I will feel ashamed to talk about my condition… I can tolerate until it is really necessary." (Interview 8, PNC, Pakistan)
	Q48	"Some women don't share. Some women keep everything inside." (Interview 11, PNC, Pakistan)
**Should not be assessed**	Q49	"Doctors should not ask these questions, no, they should not." (Interview 5, PNC, Pakistan)
	Q50	"No, doctor has no right to ask such questions." (Interview 6, PNC, Pakistan)
	Q51	"A doctor has many patients …Obviously, they don't have time for these details!" (FGD 4, ANC, Pakistan)
**Impact on family**	Q52	"If you ask about the family situation, our community is such that women will be afraid as to why these questions are being asked and they have a fear that in-laws will find out" (FGD 2, ANC, Pakistan)
	Q53	"If the doctor asks in front of our family, that will help change bad behaviours in the family" (FGD 3, ANC, Pakistan)
**Solutions**	Q54	"They should use questions in a way that are focused and clear." (FGD3, ANC, Pakistan)
	Q55	"Do you have any tension? How is your home environment? How is your husband and your family-in-laws behaviour with you?" (Interview 10, PNC, Pakistan)

For peer review only - http://bmjopen.bmj.com/site/about/guidelines.xhtml

For peer review only

For peer review only - http://bmjopen.bmj.com/site/about/guidelines.xhtml

LSTM
CENTRE FOR MATERNAL AND NEWBORN HEALTH

Topic guides for women during and after pregnancy for their views and understanding of maternal health and ill-health

For peer review only

For peer review only - http://bmjopen.bmj.com/site/about/guidelines.xhtml

1. TOPIC GUIDE FOR PREGNANT AND POSTNATAL WOMEN

PROFILE OF WOMEN

Once participants have signed the consent form, ask them to answer these following questions. It will help analyse the qualitative data.

Study setting: ____

Age: ____

Parity: ____

Pregnant status / antenatal or postnatal and number of weeks: _____________________

FORMAT

- Introduce yourself and welcome the group.
- Explain that we are here to learn more about the groups' views on their general views regarding what they understand all types of ill-health to be during and after pregnancy.
- In addition, explain that we are keen to understand what the group in general think regarding questions concerning other types of ill-health that may be a risk factor to pregnancy and how best to approach and ask these questions routinely in their setting.
- Ensure everyone has provided information for the registration log sheet and has a participant information sheet.
- Obtain informed consent for each participant.
- Provide a brief introduction to the relevant ground rules for the key informant interview or focus group discussion:
  (1) The discussion will last a maximum of 1 hour
  (2) The content of the discussion will remain confidential and will not be used outside of the group except for the purposes of the research
  (3) Participants are also requested to keep the discussion confidential after the session
  (4) Participants' names will not be used when reporting the findings
  (5) Participants are encouraged to speak their mind and there are no right or wrong answers
  (6) Participants should not hesitate to disagree with someone else
  (7) But should not talk at once
  (8) The interview will be recorded using a small hand held voice recorder device.
- A voice recorder will be used only to ensure that none of the information from the FGD is lost, and to facilitate analysis of the data from the interviews.

For peer review only - http://bmjopen.bmj.com/site/about/guidelines.xhtml

MATERIALS

Topic guide, log, notepad, pens, voice recorders, batteries, information sheet, consent form, transport reimbursement and reimbursement form, refreshments, voice recorders.

FGD QUESTION GUIDE

Reminder to moderator: listen actively and always use probing questions to follow up and obtain further detail from the group (e.g. can you tell me more about that, can you give an example, why is that).

Introduction

We are very interested to hear about your general views regarding what you understand health and ill-health to be for women that you care for during and after pregnancy.

In addition, we are keen to understand how best to ask general questions concerning other types of ill-health that may be a risk factor to the pregnancy. These general questions will include how best to screen for psychological ill-health (depression) or social ill-health (domestic violence or substance misuse) during and after pregnancy.

We would like to understand your perceptions regarding the possible barriers or enabling factors to routinely screen women during and after pregnancy for all types of ill-health (physical, psychological and social) and whether you think women's general health needs are current being met in routine antenatal and postnatal care in your setting.

General questions to ask

1. What is health and what is ill-health during and after pregnancy?

We are interested to know about your general views of what health is and what ill-health in general is for women during and after their pregnancy.

- What do women in your setting consider to be 'good health'?
- What do women in your setting you consider to be 'poor health'?
- In relation to pregnancy: what would good health be and what would poor health be?
- If we consider poor health in pregnancy, what does this mean?
- What kinds of conditions or complaints do pregnant women tend to have?

- Is poor health different in pregnancy and after pregnancy? How?

- Probe: is health only related to physical health?

For peer review only - http://bmjopen.bmj.com/site/about/guidelines.xhtml

- Probe: does ill-health during and after pregnancy also include psychological, and social factors?
- Probe: In your opinion, do women have ill-health during and after their pregnancy? and if so what how does this bother them?

2. How best can a healthcare provider ask questions routinely regarding other forms of ill-health women experience during and after pregnancy?

We are keen to understand how best you think a healthcare provider can ask routinely regarding other forms of ill-health women experience during and after pregnancy, that may be a risk factor to the pregnancy, for example psychological ill-health (depression) or social ill-health (domestic violence or substance misuse)?

Can you tell us your general view and opinion on whether it is acceptable for healthcare providers to make enquiries regarding not just physical but also psychological and social ill-health during routine antenatal and postnatal care?

- Probe: What do you think about women being asked about their perception of their health in pregnancy?
- What kinds of questions do you think pregnant women should be asked to assess how they feel about their health?

- What do you think women would think regarding being asked about their mental well-being or social factors?
- Is it acceptable to be asked about this? Why? Why not?
- How do you think women should be asked these questions?

3. We would like to understand your general perceptions regarding the possible barriers or enabling factors to routinely screen women during and after pregnancy for all types of ill-health (physical, psychological and social).

- In your community, how is pregnancy perceived?
- Are pregnant women treated any differently? In what way?
- When women are pregnant do they expect to feel ill?
- What kinds of conditions or complaints are perceived as ‘routine’ during pregnancy?
- What conditions or complaints are not considered a ‘routine’ part of pregnancy?
- How do women deal with minor common discomforts s during pregnancy?

For peer review only - http://bmjopen.bmj.com/site/about/guidelines.xhtml

- How do women deal with those that are not considered a common part of pregnancy?

4. Do you think women's general health needs are current being met in routine antenatal and postnatal care in your setting?

We would like to understand more about whether the existing health care system in your general opinion, meets the health needs of women during and after pregnancy in your community?

Probe: To what extent do you think the health facility meets womens' needs during and after pregnancy? In your opinion does the health system in general provide good quality to women during and after pregnancy?

Probe: What do you think should be done to improve provision of services for women during and after pregnancy?

In summary:
Ask if participants would like to add further comments.

Bring the meeting to a close by summarizing the main points.

Do not forget to say thank you to the participants for their time and active participation.

For peer review only - http://bmjopen.bmj.com/site/about/guidelines.xhtml

COREQ (COnsolidated criteria for REporting Qualitative research) Checklist

A checklist of items that should be included in reports of qualitative research. You must report the page number in your manuscript where you consider each of the items listed in this checklist. If you have not included this information, either revise your manuscript accordingly before submitting or note N/A.

Topic	Item No.	Guide Questions/Description	Reported on Page No.
**Domain 1: Research team and reflexivity**			
Personal characteristics			
Interviewer/facilitator	1	Which author/s conducted the interview or focus group?	
Credentials	2	What were the researcher's credentials? E.g. PhD, MD	
Occupation	3	What was their occupation at the time of the study?	
Gender	4	Was the researcher male or female?	
Experience and training	5	What experience or training did the researcher have?	
Relationship with participants			
Relationship established	6	Was a relationship established prior to study commencement?	
Participant knowledge of the interviewer	7	What did the participants know about the researcher? e.g. personal goals, reasons for doing the research	
Interviewer characteristics	8	What characteristics were reported about the inter viewer/facilitator? e.g. Bias, assumptions, reasons and interests in the research topic	
**Domain 2: Study design**			
Theoretical framework			
Methodological orientation and Theory	9	What methodological orientation was stated to underpin the study? e.g. grounded theory, discourse analysis, ethnography, phenomenology, content analysis	
Participant selection			
Sampling	10	How were participants selected? e.g. purposive, convenience, consecutive, snowball	
Method of approach	11	How were participants approached? e.g. face-to-face, telephone, mail, email	
Sample size	12	How many participants were in the study?	
Non-participation	13	How many people refused to participate or dropped out? Reasons?	
Setting			
Setting of data collection	14	Where was the data collected? e.g. home, clinic, workplace	
Presence of non-participants	15	Was anyone else present besides the participants and researchers?	
Description of sample	16	What are the important characteristics of the sample? e.g. demographic data, date	
Data collection			
Interview guide	17	Were questions, prompts, guides provided by the authors? Was it pilot tested?	
Repeat interviews	18	Were repeat inter views carried out? If yes, how many?	
Audio/visual recording	19	Did the research use audio or visual recording to collect the data?	
Field notes	20	Were field notes made during and/or after the inter view or focus group?	
Duration	21	What was the duration of the inter views or focus group?	
Data saturation	22	Was data saturation discussed?	
Transcripts returned	23	Were transcripts returned to participants for comment and/or	

For peer review only

Topic	Item No.	Guide Questions/Description	Reported on Page No.
		correction?	
**Domain 3: analysis and findings**			
Data analysis			
Number of data coders	24	How many data coders coded the data?	
Description of the coding tree	25	Did authors provide a description of the coding tree?	
Derivation of themes	26	Were themes identified in advance or derived from the data?	
Software	27	What software, if applicable, was used to manage the data?	
Participant checking	28	Did participants provide feedback on the findings?	
Reporting			
Quotations presented	29	Were participant quotations presented to illustrate the themes/findings? Was each quotation identified? e.g. participant number	
Data and findings consistent	30	Was there consistency between the data presented and the findings?	
Clarity of major themes	31	Were major themes clearly presented in the findings?	
Clarity of minor themes	32	Is there a description of diverse cases or discussion of minor themes?	

Developed from: Tong A, Sainsbury P, Craig J. Consolidated criteria for reporting qualitative research (COREQ): a 32-item checklist for interviews and focus groups. *International Journal for Quality in Health Care*. 2007. Volume 19, Number 6: pp. 349 – 357

Once you have completed this checklist, please save a copy and upload it as part of your submission. DO NOT include this checklist as part of the main manuscript document. It must be uploaded as a separate file.

For peer review only
